# Chromatin accessibility dynamics of *Chlamydia*-infected epithelial cells

**DOI:** 10.1186/s13072-020-00368-2

**Published:** 2020-10-27

**Authors:** Regan J. Hayward, James W. Marsh, Michael S. Humphrys, Wilhelmina M. Huston, Garry S. A. Myers

**Affiliations:** 1 The ithree Institute, University of Technology Sydney, Sydney, NSW Australia; 2 Max Planck Institute for Developmental Biology, Tuebingen, Germany; 3 Institute for Genome Sciences, University of Maryland School of Medicine, Baltimore, MD USA; 4 School of Life Sciences, Faculty of Science, University of Technology Sydney, Sydney, NSW Australia

**Keywords:** Chlamydial infection, *Chlamydia trachomatis*, Chromatin accessibility, FAIRE-Seq, Bacterial infection

## Abstract

Chlamydia are Gram-negative, obligate intracellular bacterial pathogens responsible for a broad spectrum of human and animal diseases. In humans, *Chlamydia trachomatis* is the most prevalent bacterial sexually transmitted infection worldwide and is the causative agent of trachoma (infectious blindness) in disadvantaged populations. Over the course of its developmental cycle, *Chlamydia* extensively remodels its intracellular niche and parasitises the host cell for nutrients, with substantial resulting changes to the host cell transcriptome and proteome. However, little information is available on the impact of chlamydial infection on the host cell epigenome and global gene regulation. Regions of open eukaryotic chromatin correspond to nucleosome-depleted regions, which in turn are associated with regulatory functions and transcription factor binding. We applied formaldehyde-assisted isolation of regulatory elements enrichment followed by sequencing (FAIRE-Seq) to generate temporal chromatin maps of *C. trachomatis*-infected human epithelial cells in vitro over the chlamydial developmental cycle. We detected both conserved and distinct temporal changes to genome-wide chromatin accessibility associated with *C. trachomatis* infection. The observed differentially accessible chromatin regions include temporally-enriched sets of transcription factors, which may help shape the host cell response to infection. These regions and motifs were linked to genomic features and genes associated with immune responses, re-direction of host cell nutrients, intracellular signalling, cell–cell adhesion, extracellular matrix, metabolism and apoptosis. This work provides another perspective to the complex response to chlamydial infection, and will inform further studies of transcriptional regulation and the epigenome in *Chlamydia*-infected human cells and tissues.

## Introduction

Members of the genus *Chlamydia* are Gram-negative, obligate intracellular bacterial pathogens responsible for a broad spectrum of human and animal diseases [1]. In humans, *Chlamydia trachomatis* is the most prevalent bacterial sexually transmitted infection (STI) [2], causing substantial reproductive tract disease globally [3], and is the causative agent of trachoma (infectious blindness) in disadvantaged populations [4]. All members of the genus exhibit a unique biphasic developmental cycle where the non-replicating infectious elementary bodies (EBs) invade host cells and differentiate into replicating reticulate bodies (RBs) within a membrane-bound vacuole, escaping phagolysomal fusion [5]. All chlamydial species actively modulate host cell processes to establish this intracellular niche, using secreted effectors and other proteins to facilitate invasion, internalisation and replication, while countering host defence strategies [6, 7]. At the end of the developmental cycle, RBs condense into EBs, which are released from the host cell by lysis or extrusion to initiate new infections [8].

Bacterial interactions with mammalian cells can induce dynamic transcriptional responses from the cell, either through bacterial modulation of host cell processes or from innate immune signalling cascades and other cellular responses [9–11]. In addition, bacterial effector proteins specifically targeting the nucleus (nucleomodulins) can influence cell physiology and directly interfere with transcriptional machinery including chromatin remodelling, DNA replication and repair [12]. Host cell epigenetic-mediated transcriptional regulatory changes, including histone modifications, DNA methylation, chromatin accessibility, RNA splicing, and non-coding RNA expression [13–15] may also be arbitrated by bacterial proteins and effectors. Consistent with host cell interactions with other bacterial pathogens, *C. trachomatis* infection alters host cell transcription over the course of its developmental cycle [16] and may also modulate the host cell epigenome. For example, NUE (NUclear Effector), a *C. trachomatis* type III secreted effector with methyltransferase activity, enters the host nucleus and methylates eukaryotic histones H2B, H3 and H4 in vitro [17]. However, the ultimate gene targets of NUE activity or the affected host transcriptional networks are uncharacterised, as is the influence of chlamydial infection on the host cell epigenome in general.

Genetic information in eukaryotes is compactly organised within the nucleus of each cell in highly ordered structures composed of DNA and proteins, designated chromatin. Cellular processes occur when chromatin fibres become less condensed, providing areas of open chromatin which allow transcription to proceed. Areas of open chromatin are associated with active DNA regulatory elements, including promoters, enhancers, silencers, and insulators. Chromatin accessibility is also relevant to alternative splicing, alternative promoter usage and alternative polyadenylation, where different forms of RNA are generated from the same gene [18]. Thus, the underlying structures (introns, exons, TSS and TTS) can be differentially used and thus differentially accessed. To examine the impact of chlamydial infection on host cell chromatin dynamics, we applied FAIRE-Seq (formaldehyde-assisted isolation of regulatory elements sequencing) [19] to *C. trachomatis*-infected HEp-2 epithelial cells and time-matched mock-infected cells, spanning the chlamydial developmental cycle (1, 12, 24 and 48 h post-infection). FAIRE protocols rely on the variable crosslinking efficiency of DNA to nucleosomes by formaldehyde, where nucleosome-bound DNA is more efficiently crosslinked. DNA fragments that are not crosslinked are subsequently enriched in the aqueous phase during phenol–chloroform extraction. These fragments represent regions of open chromatin, which in turn can be associated with regulatory factor binding sites. In FAIRE-Seq, libraries are generated from these enriched fragments, followed by sequencing and read mapping to a reference genome [19], allowing patterns of chromatin accessibility to be identified [20]. We identify infection-responsive changes in chromatin accessibility over the chlamydial developmental cycle, and identify several candidate host transcription factors that may be relevant to the cellular response to chlamydial infection. We note that the experimental design used here cannot distinguish *Chlamydia*-mediated effects from infection-specific or non-specific host cell responses. Further experiments with inactivated *Chlamydia* or selected gene knock-outs or knock-downs will help to elucidate the extent of specific *Chlamydia*-mediated interference with the host cell epigenome. We also note that the use of in vitro immortalised HEp-2 epithelial cells means that, despite their utility and widespread use in chlamydial research, the full diversity of host cell responses that are likely to be found within in vivo infections will not be captured.

## Methods

### Cell culture, infection and experimental design

HEp-2 cells (American Type Culture Collection, ATCC No. CCL-23) were grown as monolayers in 6 × 100 mm TC dishes until 90% confluent. Monolayers were infected with *C. trachomatis* serovar E in sucrose–phosphate–glutamate (SPG) buffer as previously described [21]. Additional monolayers were mock-infected with SPG only. The infection was allowed to proceed 48 h prior to EB harvest, as previously described [21]. *C. trachomatis* EBs and mock-infected cell lysates were subsequently used to infect fresh HEp-2 monolayers. Fresh monolayers were infected with *C. trachomatis* serovar E in 3.5 mL SPG buffer for an MOI ~ 1 as previously described [21], which routinely observes 95% + infectivity; centrifugation was used to synchronise infections. Infections and subsequent culture were performed in the absence of cycloheximide or DEAE dextran. A matching number of HEp-2 monolayers were also mock-infected using uninfected cell lysates. Each treatment was incubated at 25 °C for 2 h and subsequently washed twice with SPG to remove dead or non-viable EBs. 10 mL fresh medium (DMEM + 10% FBS, 25 μg/mL gentamycin, 1.25 μg/mL fungizone) was added and cell monolayers incubated at 37 °C with 5% CO_2_. Three biological replicates of infected and mock-infected dishes per time were harvested post-infection by scraping and resuspending cells in 150 μL sterile PBS. Resuspended cells were stored at − 80 °C.

### FAIRE enrichment and sequencing

Formaldehyde-crosslinking of cells, sonication, DNA extraction of FAIRE-enriched fractions and Illumina library preparation was performed as previously described [19]. Libraries were prepared in triplicate from infected and mock-infected samples at 1, 12, 24 and 48 h, using the Illumina TruSeq Sample Prep kit, and were sequenced on the Illumina 2500 platform (101 bp paired-end read protocol) at the Genome Resource Centre, Institute for Genome Sciences, University of Maryland School of Medicine. Sequence data are available from the NCBI GEO archive GSE132448.

### Bioinformatic analyses

Raw sequencing reads were trimmed and quality checked using Trimmomatic (0.36) [22] and FastQC (0.11.5) [23]. Trimmed reads were aligned to the human genome (GRCh 38.87) using Bowtie2 (2.3.2) [24] with additional parameters of ‘no mismatches’ and ‘–very-sensitive-local’. Duplicate reads were removed using Picard tools (2.10.4) [25]. Additional replicate quality control was performed using deepTools (2.5.3) [26] and in-house scripts: https://github.com/reganhayward/Manuscripts-code.

Peak calling of open chromatin regions was performed using MACS2 (2.1.1) [27] in paired-end mode, with additional parameters of ‘–no-model –broad –q 0.05′ and MACS2 predicted extension sizes. Care was taken to ensure parameters were best suited for FAIRE-seq data, particularly as peaks are generally broader than other methods as well as exhibiting a slightly higher background signal [28]. All replicates were called separately, with significant peaks determined against the software-predicted background signal. Any peaks that fell within ENCODE blacklisted regions (regions exhibiting ultra-high signal artefacts) [29], or were located on non-standard chromosomes such as (ChrMT and ChrUn) were removed. At this point, we chose a trade-off between sequencing depth and retaining a higher number of replicates for each condition. This was due to a combination of stringent filtering steps and discarding any chlamydial mapped reads, resulting in medium to low coverage across the human genome. By not merging replicates, we acknowledge the loss of sequencing depth, but gain robust results through the creation of consensus peak sets which focused our analysis on reoccurring peaks/regions.

Consensus peak sets were created by combining significant peaks from the infected and mock-infected replicates for each time using Diffbind [30]. Peaks were removed if they appeared in less than two replicates. Reads were counted under each peak within each consensus peak set; the resulting read depths were normalised to their relative library sizes. The resulting count matrices from each consensus peak set were subsequently used to examine differences in chromatin accessibility between infected and mock-infected replicates at each time using the built in DESeq2 method of Diffbind (FDR < 0.05). This created a list of differential chromatin-accessible regions with fold-changes relative to the mock-infected conditions. While FAIRE enrichment is designed to recover open chromatin regions, our time-matched infected and mock-infected experimental design enables corresponding patterns of closed chromatin to be inferred by comparison between matched regions with negative fold-changes. Furthermore, we make the assumption that open and closed chromatin are directly associated with an increase or decrease in gene expression, respectively. As these data are a snapshot of infection events, this may not capture the dynamism of the infection response. For example, some regions of open chromatin may be in the process of being closed. Additionally, open chromatin regions may be facilitating the binding of transcriptional repressors, resulting in decreased expression.

Annotation of the set of differential chromatin-accessible regions was performed with Homer (v4.9) [31] and separated into three main categories: Intragenic, Promoter and Intergenic. Intergenic: located > 1 kbp upstream of the transcriptional start site (TSS), or downstream from the transcription termination site (TTS); Promoter: located within 1kbp upstream or 100 bp downstream of the TSS (all promoter regions taken from RefSeq); and, intragenic: annotated to a 3′UTR, 5′UTR, intron, exon, TTS, miRNA, ncRNA or a pseudogene. When regions overlapped multiple features, the resulting annotation was ordered by promoter, intragenic feature, then intergenic regions. To identify enhancers, all intergenic regions were compared against enhancer regions from HeLa cells using Hacer [32], Enhancer-atlas [33] and dbSuper [34]. The use of Hacer allowed enhancers from ENCODE and FANTOM5 to also be used. All enhancer regions were converted from hg19 to hg38 using the UCSC LiftOver tool [35].

Results within this manuscript show that 49% of the differently chromatin-accessible regions enriched in this chlamydial infection data are intergenic. Additional prediction-based software analyses would likely reduce the number of intergenic regions by predicting additional features such as silencers, insulators and possibly more enhancers. Although extremely useful in the right setting, we chose not to run prediction-based tools as we wanted the results to be less speculative and only highlight significant infection-relevant events.

Bimodality coefficients for the frequency distribution plots were calculated using the R-package Modes [36]. Long non-coding RNAs (lncRNA) were identified using data from HeLa cells [37], using a screen score > 2.

Motif analysis was performed with Homer [31]. Target sequences were regions with significant differential chromatin accessibility as identified by DESeq 2, while the number of background sequences were software-determined, randomly selected regions throughout the human genome (excluding target regions and normalised for GC content). Additional parameters included using a hypergeometric distribution, searching for motifs between 8- and 16-bp long and allowing for four mismatches. To confirm motif significance within the 120 conserved regions, the number of background sequences was varied, and only motifs that appeared across a consensus of values were retained. Motif enrichment was also performed with Homer [31], followed by filtering and bioinformatic assessment of human tissue specificity where possible. Time-specific TF filtering was applied by using parameters of *p* value < 0.001 and > 5% of target sequences. For the 120 conserved regions across all times, TFs were filtered based on a *p* value < 0.05. For significant de novo TFs, motif matrices were compared against the Jaspar [38] and TomTom [39] databases Enriched TF motifs were retained only if the Homer annotation matched top hits in Jaspar or TomTom, and were also human tissue specific. Furthermore, due to the strict filtering and application of thresholds to determine the final set of TF motifs, it is possible that TFs with weak binding sites that are infection relevant may be missed.

All identified TFs from the conserved and time-specific regions were compared to publicly available gene expression data to ensure that each TF could be expressed in HEp2 cells (unpublished data from dual RNA-Seq of *C. trachomatis* serovar E in HEp-2 cells over time). For infection relevance, TF expression was also examined across a range of different times (0.5, 1.5, 3, 6, 12, 24, 30 and 48 h), cell lines (HEp2, HeLa and End1) and across different *C. trachomatis*-based infection models [40–42] and (unpublished data as described above).

When only small numbers of genes were recovered, enrichment was performed manually using publicly available databases, including NCBI [43], Uniprot [44], WikiGenes [45] and GeneCards [46]. Gene Ontology [47] analysis was performed on the 48-h time-specific differential chromatin regions as the higher number of input genes enabled significance. In-house scripts and code used throughout this manuscript are available at https://github.com/reganhayward/Manuscripts-code.

## Results and discussion

### Chromatin accessibility landscapes of *Chlamydia*-infected and mock-infected cells

**Fig. 1:**
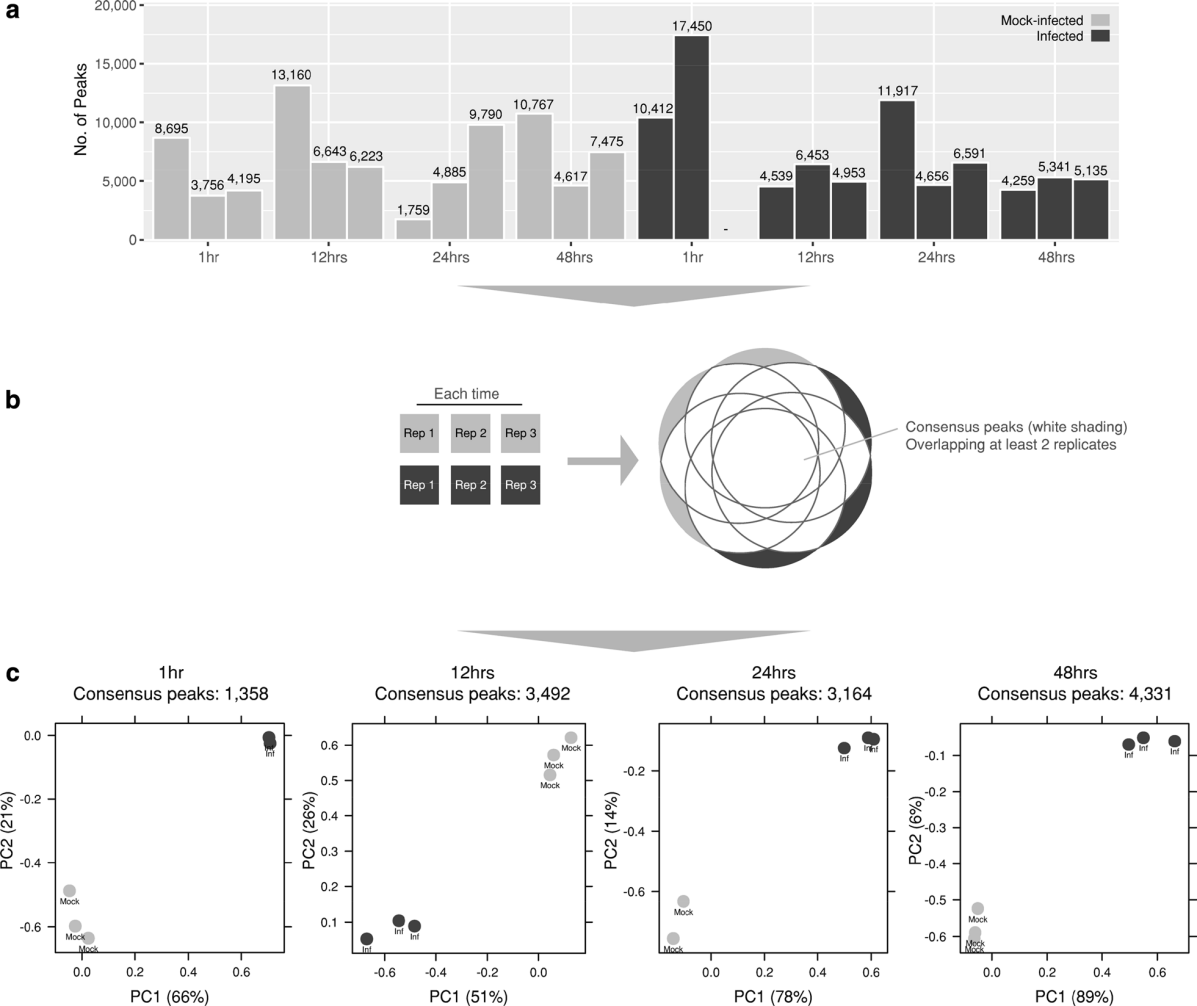
Identifying significant peaks and creating consensus peaksets. **a** Significant peaks per replicate (*p* value < 0.05). **b** Consensus peaks were created for each time by combining significant peaks from *Chlamydia*-infected and mock-infected conditions, retaining peaks which appeared in > 2 replicates. **c** PCA plots demonstrating tight clustering within each consensus peak set grouping infected and mock-infected replicates

We applied FAIRE-Seq to *C. trachomatis* serovar E-infected and mock-infected human HEp-2 epithelial cells in triplicate at 1, 12, 24, and 48 h post-infection (hpi). Following initial quality control measures, a single *C. trachomatis*-infected replicate was identified as an outlier and was removed from further analysis. Significant peaks, representing regions of open chromatin in either the mock-infected or infected conditions, were identified from reads mapped to the human genome; with 52,584,839 mapped reads for mock-infected replicates and 98,802,927 mapped reads for *Chlamydia*-infected replicates (151,387,766 in total) (Additional file 1). Each peak file was then examined in IGV to ensure peaks were dispersed genome-wide without discernible chromosomal biases (Additional file 2). The total number of significant peaks from each replicate varied across the examined times and conditions, ranging between 1759 and 17,450 peaks (Fig. 1a).

Diffbind [30] was used to group and filter peaks at each time post-infection by removing regions with low coverage, or any regions not represented across a consensus of replicates (Fig. 1b). After normalisation for library size, principal component analysis (PCA) of the consensus peak sets led to the removal of one further outlier at 24 h (mock-infected). The remaining peak sets exhibit tight clustering between mock-infected and infected conditions, respectively, at each time (Fig. 1c). Total consensus peak numbers increased across the chlamydial developmental cycle, independent of the total mapped reads over time.

### *C. trachomatis* infection is associated with temporal changes to chromatin accessibility in host cells

**Fig. 2:**
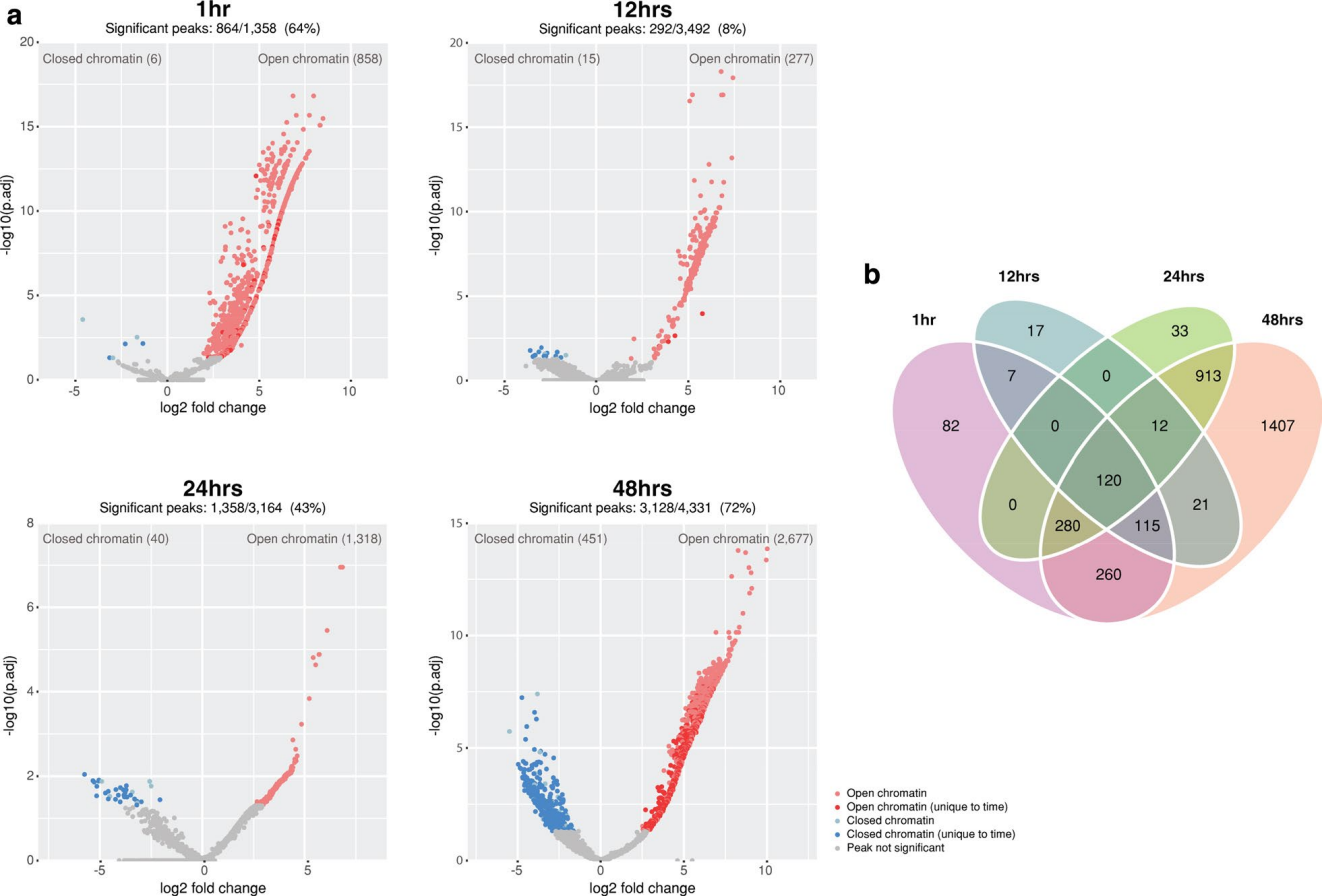
Changes in chromatin accessibility over the chlamydial developmental cycle. **a** Volcano plots highlighting changes in chromatin accessibility between infected and mock-infected conditions. Regions of closed chromatin are represented as blue dots, while open chromatin regions are red dots. Peaks unique to a specific time have darker shading. Percentages above the plots show the proportion of consensus peaks with significant changes of chromatin accessibility between conditions (FDR < 0.05). **b** Unique and conserved regions of differential chromatin accessibility across the developmental cycle

We identified genomic regions with significant differences in chromatin accessibility between infected and mock-infected conditions throughout the development cycle (FDR < 0.05). The resulting set of differential chromatin-accessible regions identifies both open and closed chromatin (relative to mock-infection). The total number of significant differentially accessible regions rose over the development cycle, with the number of regions increasing (3.6×) from 1 hpi (864) to 48 hpi (3128) (Fig. 2a). Open chromatin regions predominate over closed chromatin regions at each time (86–99%), suggesting that host cell transcription and regulatory activity increases in response to infection. Closed chromatin regions also increase over time, but at a much lower frequency. This may be an artefact of FAIRE enrichment, which is designed to specifically recover open chromatin.

**Fig. 3:**
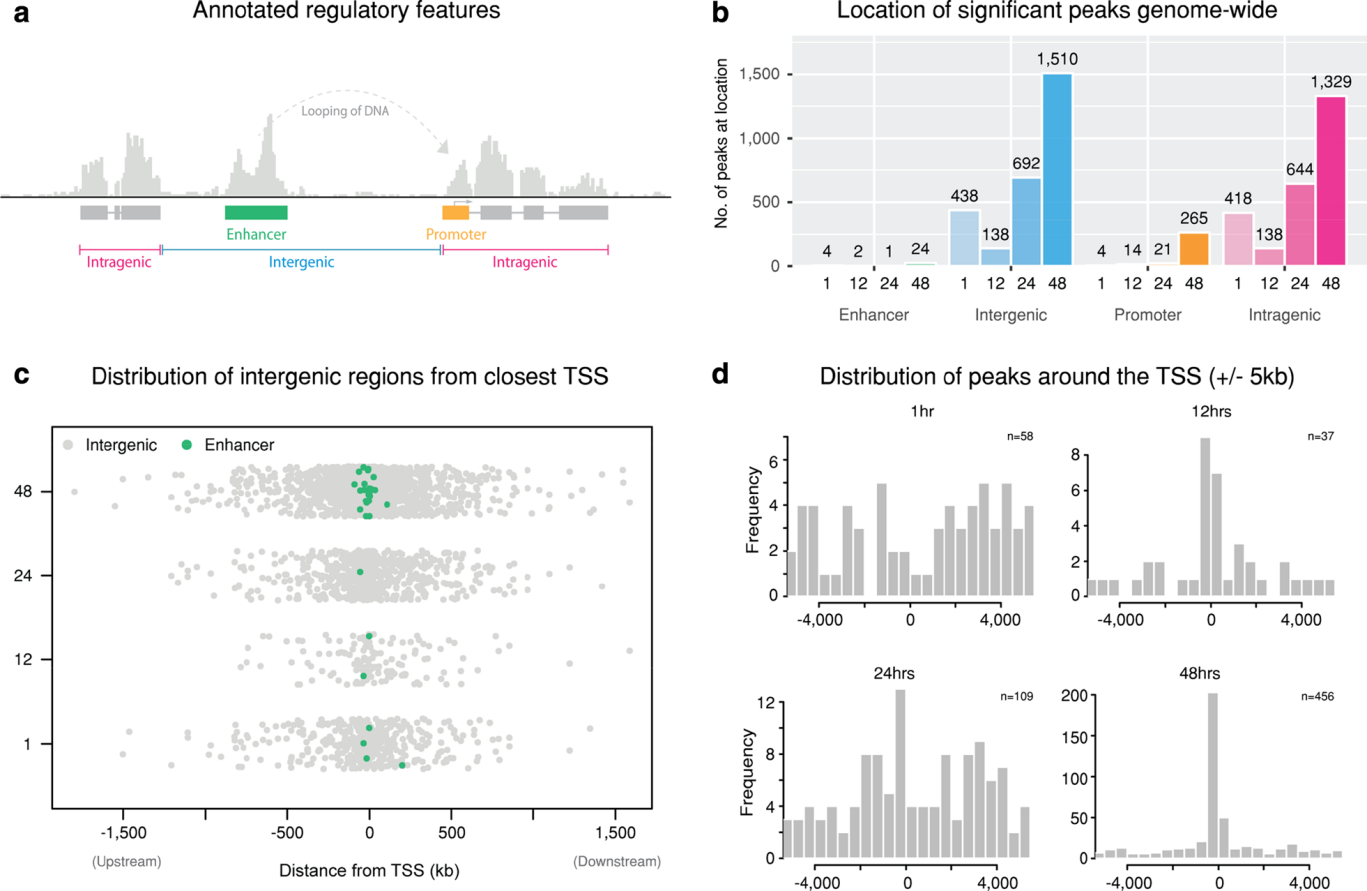
Annotation of significant peaks. **a** Example illustration of annotating significant differential peaks to enhancer, promoter, intragenic or intergenic regions. **b** Number of peaks per annotated category, separated by time. **c** All intergenic peaks plotted based on their proximity to the TSS of the closest gene. All enhancers were identified from within these regions and are coloured green. **d** Frequency distribution of significant peaks and proximity to the TSS of their associated genes (± 5 KB)

At 12 h, the number of significant differentially accessible regions was lower (8%), compared to the other times (64% at 1 hpi, 43% at 24 hpi and 72% at 48 hpi). The number of mapped reads was similar for all 12-h replicates across conditions, and similar to other times, suggesting minimal bias from the variability of the underlying mapped reads (Additional file 1) and significant peaks (Fig. 1a). In addition, each replicate had consistent peak coverage across the human genome (Additional file 2). Furthermore, the 12-h peak annotation is similar to other times (Fig. 3b), and the distribution of peaks around the TSS (Fig. 3d) are within promoter regions, as also seen at 48 h (Fig. 3d). Thus, in the absence of any discernible bias, the lower number of significant differentially accessible regions at 12 h may reflect a lower efficiency of formaldehyde-crosslinking, or that this time in the course of chlamydial infection is relatively quiescent.

120 differentially accessible chromatin regions are common at all examined times (Fig. 2b), indicating a conserved response to chlamydial infection-associated events or general disruption of cellular homeostasis, irrespective of infection progression. In addition, unique sets of differentially accessible regions are found at each time post-infection, also highlighting the dynamism of the cellular response to infection over time, particularly at 48 hpi (Fig. 2b). Differential chromatin-accessible regions were annotated based on four categories (intragenic, enhancer, promoter and intergenic) as described in the Methods, and portrayed in Fig. 3a. It should be noted that although the enhancer region displayed in the figure is upstream of an associated promoter, they can appear anywhere throughout the genome. Enhancers often interact with genes through looping of DNA (Fig. 3a), but can also interact through tracking, linking and relocation mechanisms [34]. Most infection-associated differential chromatin-accessible regions were annotated to either intergenic or intragenic regions (Fig. 3b). Intergenic regions spanned considerable distances upstream and downstream from the closest gene (Fig. 3c), while enhancers that were identified from within these regions appear much closer to the TSS. Intragenic regions were predominantly (> 90%) annotated to intronic regions (Additional file 3), consistent with other chromatin accessibility studies [48, 49], and the overall distribution of protein-coding genes within the human genome [50]. The distribution of differential chromatin-accessible regions around TSSs (± 5 kb) at 12 and 48 hpi show that many regions are in close proximity to TSSs, with many regions directly upstream. Due to the strict classification of overlapping RefSeq–based promoters (− 1000 to 100 bp from TSS) employed here, we are confident these regions represent infection-relevant activity. At 24 hpi we also see a large number of regions directly upstream of the TSS, but also an increase of regions and variability further up and downstream. At 1 hpi, the regions exhibit a slight bi-modal distribution (bimodality coefficient 0.67), with fewer regions directly surrounding the TSS (Fig. 3d). The increased number of regions not immediately surrounding TSSs at 1 and 24 hpi may suggest additional regulatory mechanisms such as different transcription initiation sites, or that differential intron/exon usage may be contributing to, or otherwise influencing chromatin accessibility upon chlamydial infection.

### Differential chromatin accessibility at promoter regions

**Fig. 4:**
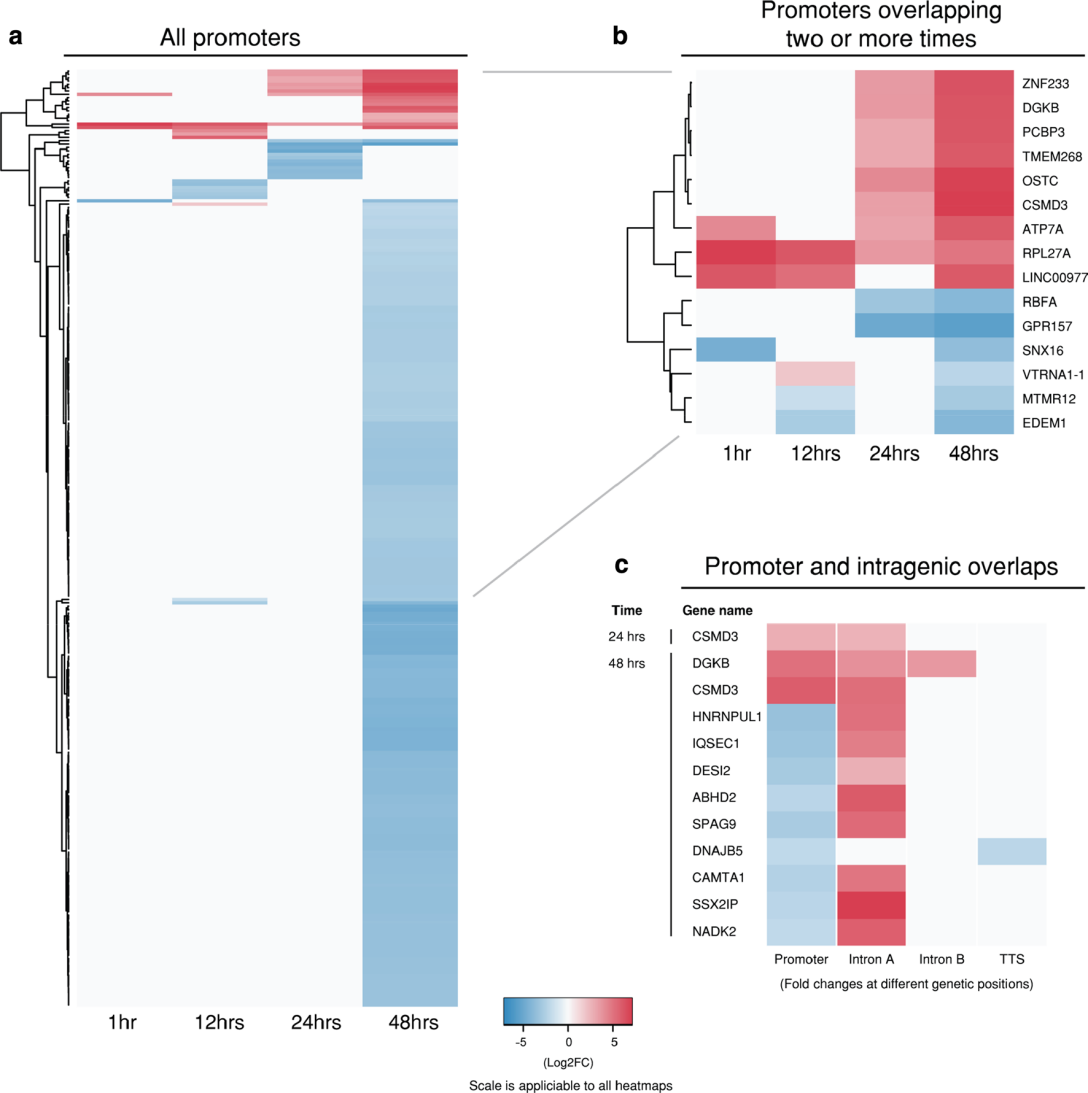
Differential chromatin accessibility within promoter regions. Heatmaps of significant differential peaks that were annotated to a promoter region. A All promoter regions from each time post-infection. B Promoters overlapping two or more different time points post-infection. Red and blue shading indicates fold-changes, while grey indicates no significant peaks. C Genes which contained significant differential peaks within a promoter region and also within one or more intragenic regions

The proportion of all differentially accessible regions mapping to promoter regions is 4 (0.5%) at 1 hpi, 14 (4.8%) at 12 hpi, 21 (1.5%) at 24 hpi and 265 (8.5%) at 48 hpi (Fig. 4a). Notably, 48 hpi exhibits a > tenfold increase in the number of significant regions compared to 24 hpi, with the majority of regions showing a reduction in chromatin accessibility, likely representing downregulation of the associated genes (Fig. 4a). The large number of differentially accessible chromatin regions within promoters at 48 h is a likely reflection of the diversity of events occurring at this late stage of the developmental cycle, including apoptosis, necrosis, lysis and cellular stress. Associated 48 hpi genes are linked with heat-shock stress (*DNAJB1*, *DNAJB5*, *DNAJC21* and *HSPA1B*), cell defence (*ILF2, MAP2K3* and *STAT2*), and cell stress/apoptosis (*ATF3*, *PPM1B*, *GAS5*, *BAG1* and *TMBIM6*). *ATP7A*, which has a promoter exhibiting an increase in chromatin accessibility, is a key regulator of copper transport into phagosomes as part of a host cell response to intracellular infection [51, 52].

Fifteen promoter-specific differentially accessible regions are found at two or more times. Two promoter regions are associated with genes encoding sorting nexin 16 (*SNX16*) and oligosaccharyltransferase complex subunit (*OSTC*), respectively (Fig. 4b). The promoter region of *OSTC* exhibits increased chromatin accessibility at 24 and 48 h; *OSTC* is linked to cellular stress responses [53]. Conversely, *SNX16* shows a reduction in chromatin accessibility at both 1 and 48 hpi. Sorting nexins are a family of phosphatidylinositol-binding proteins sharing a common PX domain that are involved in intracellular trafficking. Sorting nexins are a key component of retromer, a highly conserved protein complex that recycles host protein cargo from endosomes to plasma membranes or the Golgi [54]. Retromer is targeted by several intracellular pathogens, including *Chlamydia*, as a key strategy for intracellular survival [55]. The *C. trachomatis* effector protein, IncE, binds to sorting nexins 5 and 6, disrupting retromer-mediated host trafficking pathways [55] and potentially perturbing the endolysomal-mediated bacterial destruction capacity of the host cell [56]. However, *SNX16* is a unique member of this family, containing a coiled-coil domain in addition to a PX domain, and is not associated with retromer [57]. *SNX16* is instead associated with the recycling and trafficking of E-cadherin [57], which mediates cell–cell adhesion in epithelial cells, and is associated with a diversity of tissue-specific processes, including fibrosis and epithelial–mesenchymal transition (EMT) [58]. Separately, *C. trachomatis* infection has been shown to downregulate E-cadherin expression via increased promotor methylation, potentially contributing to EMT-like changes [59]. Thus, downregulation of *SNX16*, as inferred by the observed reduction in promotor-associated chromatin accessibility may contribute to chlamydial fibrotic scarring outcomes. In other bacterial pathogens, modulation of E-cadherin is a known virulence mechanism where it is degraded by proteases, such as HtrA, disrupting tight and adherens junctions to facilitate invasion through the epithelial barrier [60, 61]. Although chlamydial HtrA has been detected outside the inclusion and in exported blebs [62], E-cadherin has not yet been identified as a chlamydial HtrA target. Nevertheless, HtrA has been shown to be critical for in vivo chlamydial infections, indicating that this functionality may be revealed in the future [63].

Genes in immediate proximity to the promoter-specific differentially accessible chromatin regions were compared to genes containing differentially accessible intragenic peaks (Fig. 4c). Of the 12 genes identified, only one (*CSMD3*) appeared at more than one time point post-infection. All genes exhibited changes in chromatin accessibility at intronic regions, except *DNAJB5*, which exhibited an increase in chromatin accessibility at its promoter and TTS. *DGKB* was the only gene to exhibit changes in chromatin accessibility at its promoter and at more than one intronic region, each with decreased accessibility. *DGKB* is a diacylglycerol kinase that metabolises 1,2-diacylglycerol (*DAG*) to produce phosphatidic acid (*PA*), a key precursor in the biosynthesis of triacylglycerols and phospholipids, and a major signalling molecule [64]. *Chlamydia* obtains and redirects host-derived lipids through multiple pathways [65].

### Differential chromatin accessibility from enhancer-regulated genes

**Fig. 5:**
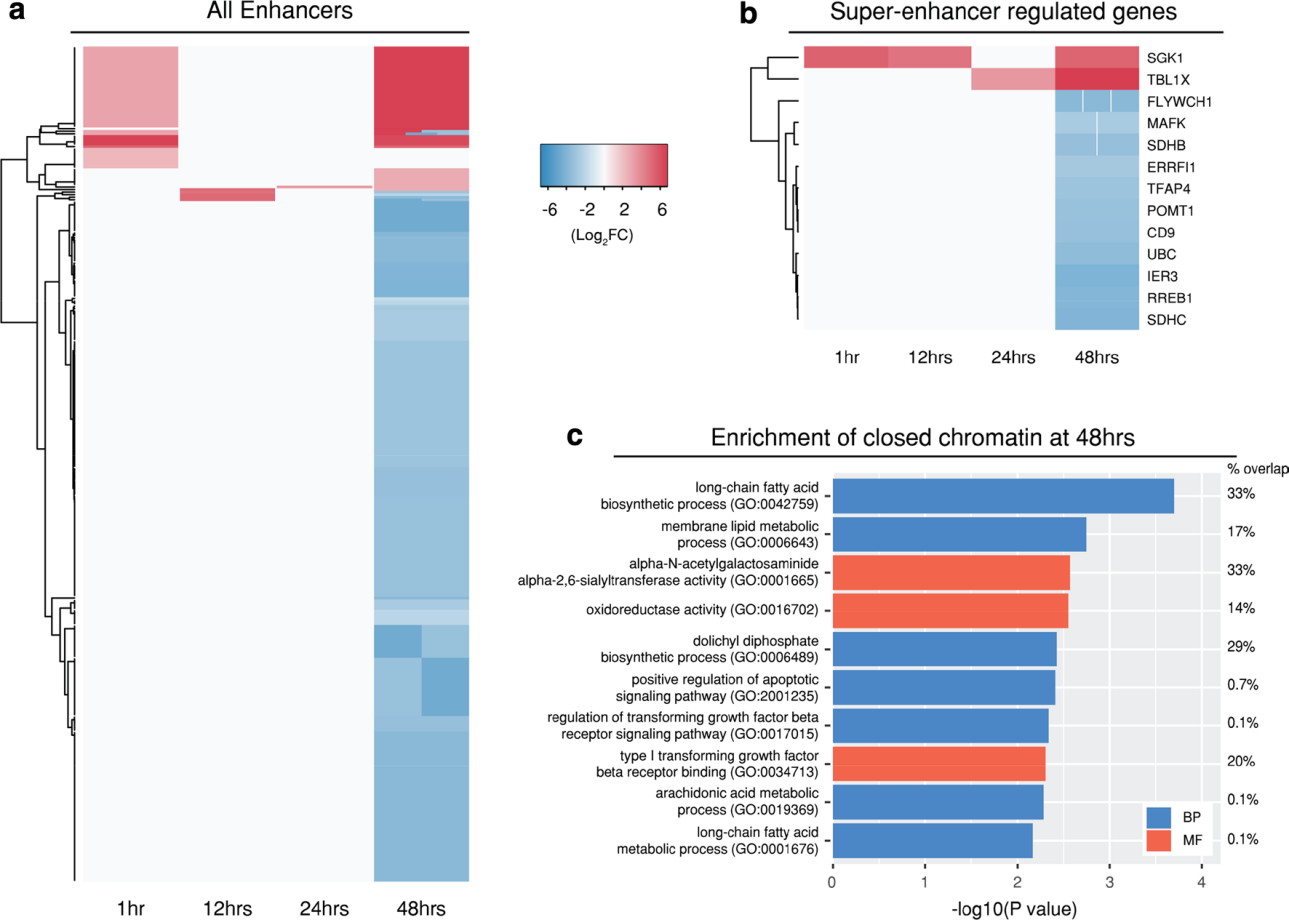
Differential chromatin accessibility within enhancer regions. Significant differential peaks annotated as intergenic were compared against tissue-specific enhancers. **a** All enhancer regions across each of the four times. **b** Super-enhancer regulated genes. Red and blue shading indicate fold-changes, while grey indicates that no significant peaks were associated with that enhancer at that time. Some enhancer regions contain more than one peak, explaining why there are multiple fold-changes at some times. **c** Gene Ontology enrichment from the large number of enhancer-associated genes at 48 h. BP and MF correspond to biological process and molecular function

Changes in chromatin accessibility of regions overlapping tissue-specific transcriptional enhancers from a range of online databases were examined, identifying 316 enhancer and 13 “super-enhancer” regulated genes (Fig. 5a, b). The super-enhancers used are defined as clusters of transcriptional enhancers that drive cell-type-specific gene expression, are crucial to cell identity, and can contain disease-associated sequence variations [66]. Each enhancer can regulate more than one gene, explaining the substantial increase in enhancers (Fig. 3b). The majority of super-enhancers exhibited an decrease in chromatin accessibility, and were associated with genes mediating energy production (*SDHB* and *CDHC*), cell protection (*IER3*) and the stress-regulated polyubiquitin gene *UBC* (ubiquitin C) [67] that is directly associated with ubiquitination, which is discussed in further detail below. Only one super-enhancer regulated gene appeared across three times post-infection (*SGK1* at 1, 12 and 48 h), with an increase in chromatin accessibility. *SGK1* (serum/glucocorticoid regulated kinase) is associated with a range of different cellular processes that are crucial to reproductive activities, with deregulation resulting in reproductive disorders such as pregnancy loss, infertility and endometriosis [68].

The majority (78%) of enhancer regions were found at 48 h post-infection, and predominantly exhibited decreased chromatin accessibility (Fig. 5c). GO analysis of closed chromatin regions identified the biological process *“long*-*chain fatty acid biosynthetic process (GO:0042759)”* with the greatest significance (−log10 *p* value 3.7). Decreased availability of long-chain fatty acids such as lauric acid and capric acid can inactivate *C. trachomatis* [69]. Reduced expression of other long-chain fatty acids such as oleic acid also negatively impacts growth and maintenance of the chlamydial inclusion membrane [70].

Four putative enhancer-regulated genes were further identified (*CROCCP2*, *LA16c*-*321D4.2*, *LINC00514* and *RP5*-*1173A5.1*) by comparing chromatin accessibility changes that mapped to lncRNAs affecting cell growth and proliferation in HeLa cells [37]. Each region exhibited decreased chromatin accessibility (Log_2_FC ranging between − 3.3 and − 4.6). Due to the relative lack of annotation of many lncRNAs, assigning function bioinformatically is challenging. However, these lncRNAs have been found to directly impact the survival of HeLa cells [71, 72].

### Conserved host responses to infection over the chlamydial developmental cycle

**Fig. 6:**
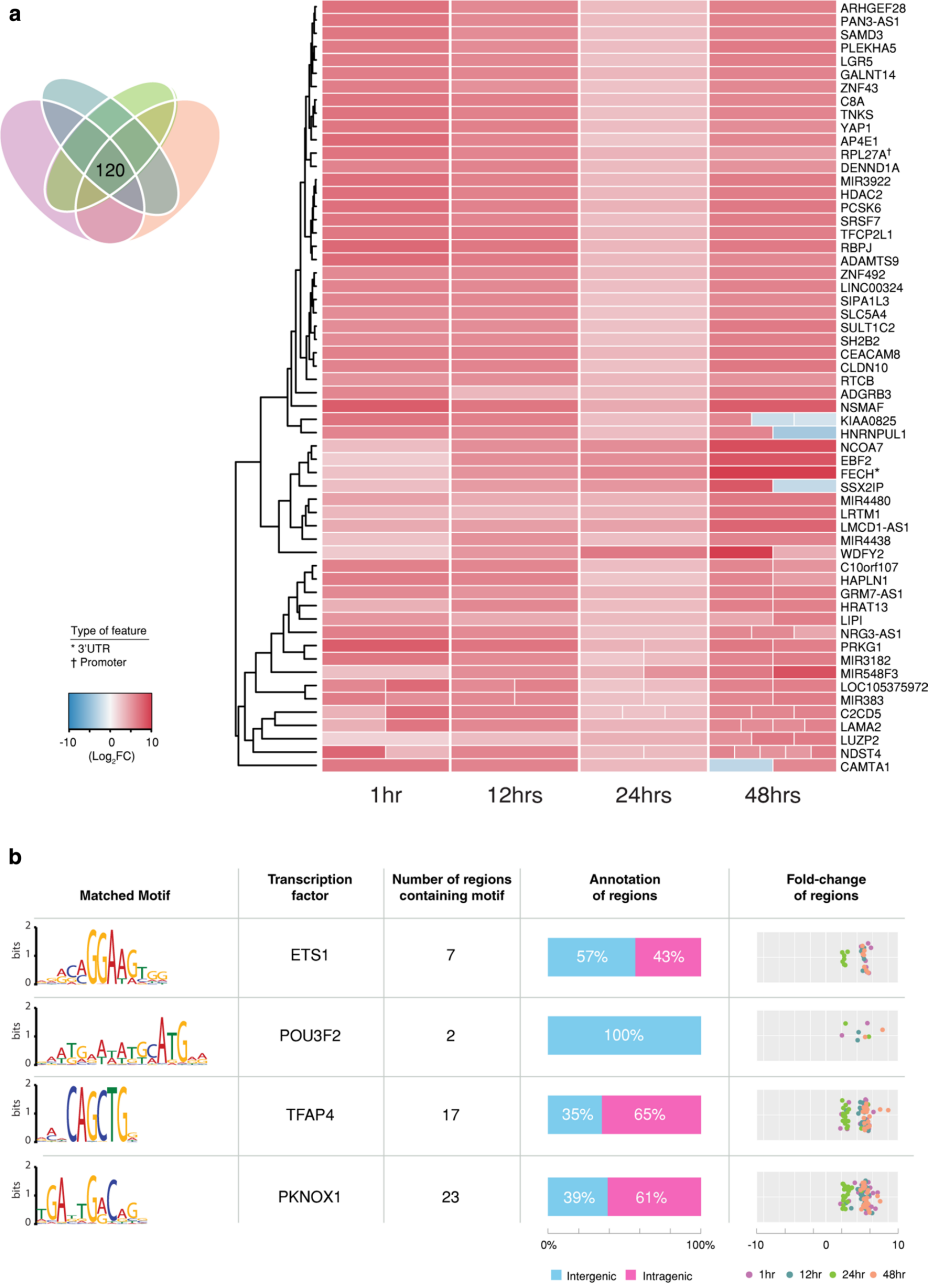
Conserved host cell response to infection. **a** 120 differentially accessible regions found in all four times were extracted, representing a conserved host cell response to infection. Intergenic regions were removed due to the ambiguity of annotating to the closest feature. If a gene contained more than one peak within a specific time, the different fold changes are split out evenly within the column at that time. **b** Significant motifs, enriched transcription factors (TFs) and associated information based on the associated chromatin accessibility within these conserved regions

The differential chromatin-accessible regions that are present at all four times during infection demonstrate a conserved host cell response to chlamydial infection (Fig. 2b). Time-specific differential chromatin accessibility is also evident over the chlamydial developmental cycle (Fig. 2b). To investigate the conserved host cell response, we focused upon 58 of the 120 differential chromatin-accessible regions (intragenic, promoter or enhancer regions) identified above, excluding the likely ambiguous intergenic regions that arise due to the ambiguity of annotating to the closest feature (Fig. 6a). 56 were within intronic regions, one within a 3′UTR (*FECH*) and one within a promoter region (*RPL27A*). Only 5 of these 58 significant differentially accessible regions show a decrease in overall chromatin accessibility. However, these same regions also exhibit increased chromatin accessibility at different intragenic locations at 48 hpi, further highlighting the potential for infection-related alternative splicing mechanisms (Fig. 6a). The remaining conserved differentially accessible regions were associated with genes involved in infection-relevant cellular processes, including *C8A* as part of the complement cascade, and lipase activity from LIPI that is essential for chlamydial replication [73]; moreover, multiple genes (*HDAC2*, *HNRNPUL1*, *NCOA7* and *YAP1*) are known transcriptional regulators [74–77]. We also examined any differential chromatin-accessible regions that appeared across three times, which identified further putative effects of chlamydial infection on the complement cascade. Key components of the membrane attack complex (MAC) and complement activation pathways exhibit increased differential chromatin accessibility (*C8B* at 1, 12 and 24 h and *CFHR5* at 24 and 48 h). Conversely, *C6* exhibits decreased chromatin accessibility at 48 h.

All conserved differentially accessible regions were also examined for known transcription factors (TF) motifs in order to identify any potential master regulators of infection responses (Fig. 6b). Four TFs were identified (*ETS1*, *POU3F2*, *TFAP4* and *PKNOX1*), represented by putative motifs within 49 different intergenic and intragenic regions. An increase in chromatin accessibility was seen at all binding sites and across all time points. *TFAP4* (Transcription Factor AP-4) functions as an activator of gene-expression of both cellular and viral genes during cellular differentiation [78]. *ETS1* (ETS Proto-Oncogene 1, Transcription Factor) also functions as an activator and is able to directly control expression levels of cytokine and chemokine genes [79, 80]. Each of these TFs have binding sites that fall within un-annotated intergenic regions. For each of the four TFs above, we further examined all significant differentially accessible chromatin regions that contained the associated motif. Regions that mapped to intergenic features were then compared to publicly available gene expression data from different *C. trachomatis*-based infection settings, as described in the methods (Additional file 4). *ETS1*, *TFAP4* and *PKNOX1* demonstrate decreases in expression across nine distinct datasets, with the greatest changes occurring at 3 h post-infection.

### Time-specific host responses to infection over the chlamydial developmental cycle

**Fig. 7:**
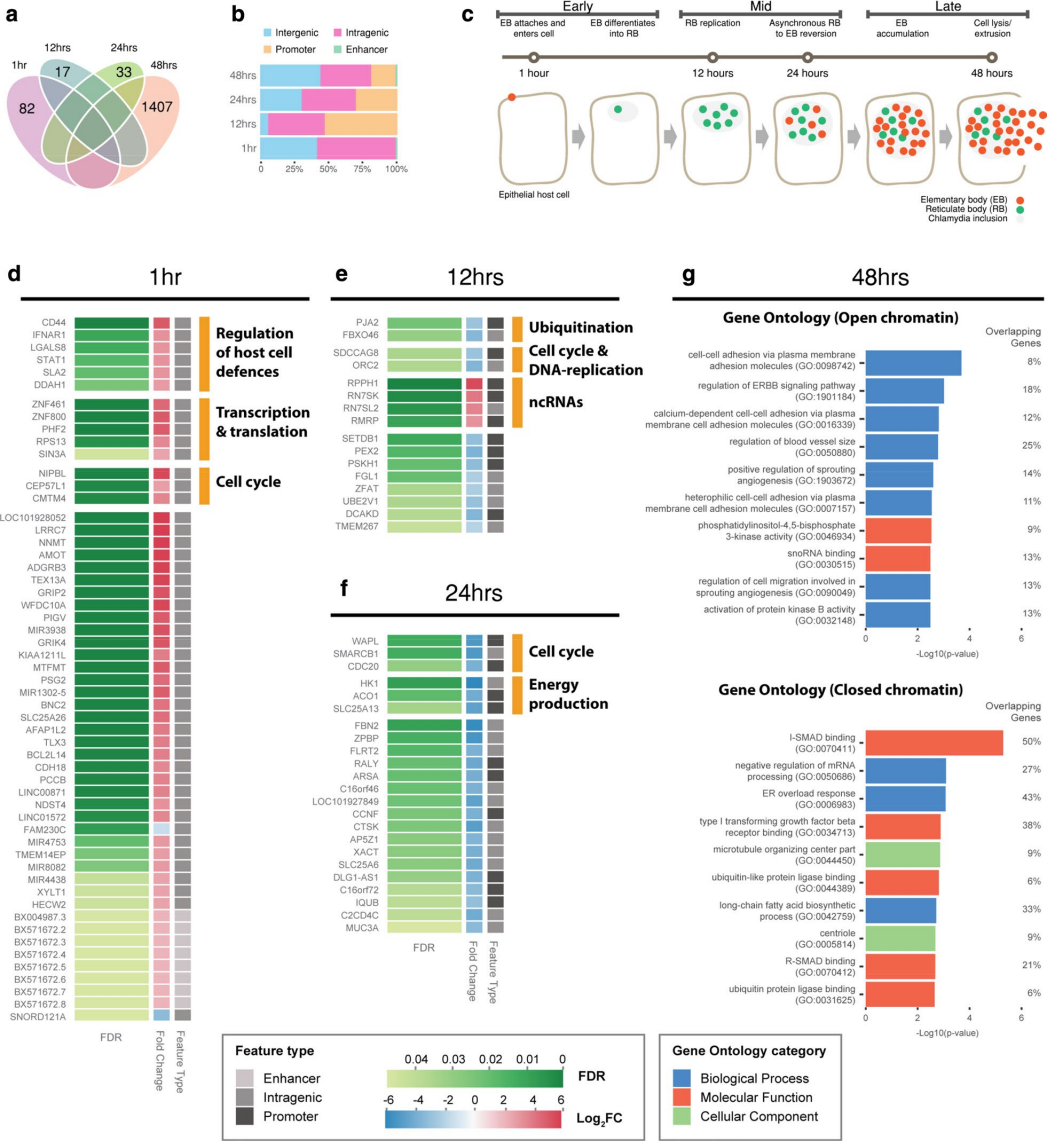
Enrichment of time-specific differential chromatin regions. **a** The numbers of significant differential chromatin-accessible regions at each time. **b** The annotation of each of these regions. **c** The in vitro *C. trachomatis* developmental cycle separated into three stages, representing known biological events from the times that were examined. **d** Annotated time-specific differential chromatin regions associated with 1 h (**d**), 12 h (**e**) and 24 h (**f**). Where genes have been grouped into annotated categories, multiple underlying sources were used for verification. **g** At 48 h, a substancial increase in genes allowed Gene Ontology (GO) enrichment. All three GO categories were enriched, with the top ten *p* values across the categories displayed

We identified unique differentially accessible chromatin regions across the chlamydial developmental cycle (Fig. 7a). At 1, 12 and 24 hpi, there are a relatively small number of significant differential chromatin-accessible regions. In contrast, 48 hpi exhibits over 1400 regions, likely reflecting the diverse processes associated with the end of the in vitro developmental cycle. As above, we focused on differential chromatin accessibility within promoters, enhancers and intragenic regions at each time: 50 at 1 hpi, 17 at 12 hpi, 27 at 24 hpi and 866 at 48 hpi (Fig. 7b, Additional file 5). We illustrate the in vitro *C. trachomatis* developmental cycle over three stages (early, mid and late), giving a broad visual representation of known biological events (Fig. 7c). Due to the limited number of differentially accessible chromatin regions at the first three times, all associated genes at those times were manually annotated, as described earlier.

At 1 hpi, increased chromatin accessibility was associated with a variety of genes involved in the regulation of host cell defences (*CD44*, *IFNAR1*, *LGALS8*, *STAT1*, *SLA2* and *DDAH1*), transcription and translation (*ZNF461, ZNF800, PHF2, RPS13* and *SIN3A*), the cell cycle (*NIPBL, CEP57L1* and *CMTM4*) and *BCL2L14* (apoptosis facilitator Bcl-2-like protein 14) a member of the Bcl-2 Family of proteins that are linked to apoptosis [81] (Fig. 7d). At 12 h, four ncRNAs were identified (*RPPH1*, *RN7SK*, *RN7SL2* and *RMRP*) that are involved in RNA processing, signalling and transcriptional regulation [82–85]. The remaining genes at 12 h exhibited decreased chromatin accessibility, encompassing the cell cycle and DNA replication (*SDCCAG8* and *ORC2*), and ubiquitination (*PJA2* and *FBXO46*) (Fig. 7e). At 24 h, all genes were associated with decreased chromatin accessibility and were grouped into two sub-categories: cell cycle (*WAPL*, *SMARCB1* and *CDC20*) and energy production (*HK1*, *ACO1* and *SLC25A13*) (Fig. 7f).

### Increased changes to differential chromatin accessibility at the end of the developmental cycle

The large number of genes associated with differential chromatin accessibility at 48 h permitted GO enrichment analysis to be performed, with the underlying genes distinguished by increased chromatin and decreased chromatin accessibility (Fig. 7g). Significantly enriched ontologies associated with regions of increased chromatin accessibility include the ErbB signalling pathway *(GO:1901184)*, which is linked to a wide range of cellular functions including growth, proliferation and apoptosis. ErbB transmembrane receptors are also often exploited by bacterial pathogens for host cell invasion [86]. Notably, epidermal growth factor receptor (EGFR), a member of the ErbB family, is the target receptor for *C. pneumoniae* Pmp21 as an EGFR-dependent mechanism of host cell entry [87]. The *C. trachomatis* Pmp21 ortholog, PmpD, also has adhesin-like functions [88], however the host ligands are unknown. Nevertheless, EGFR inhibition results in small, immature *C. trachomatis* inclusions, with calcium mobilisation and F-actin assembly disrupted [89], indicating the functional importance of EGFR and the ErbB signalling pathway for *C. trachomatis* attachment and development.

Three enriched biological processes share the term ‘*cell*–*cell adhesion* via *plasma membrane adhesion molecules*’ (*GO:0098742*, *GO:0016339* and *GO:0007157*). Several genes common to these categories with infection-responsive differential chromatin accessibility are associated with cadherins (*CDH4*, *CDH12*, *CDH17*, *CDH20*, *FAT4* and *PTPRD*), which are calcium-dependent transmembrane glycoproteins associated with the actin cytoskeleton and an essential structural component to maintain cells bind together [90]. Disruption of cadherin function has been described in *C. trachomatis* infection, and is linked to the alteration of adherens junctions and the induction of epithelial–mesenchymal transition (EMT) events that may underlie chlamydial fibrotic outcomes [59, 91]. Altered chromatin accessibility for a further cadherin-relevant locus was apparent in the promoter region of *SNX16* (see above), suggesting that alteration or disruption of cadherin regulation is a key feature of chlamydial infection. The lipid-based ontology ‘*Membrane lipid biosynthetic process* (*GO: 0030148*)’ was also associated with regions of open chromatin. *Chlamydia* scavenges a range of host cell-derived metabolites for intracellular growth and survival, particularly lipids [92, 93].

Significantly enriched ontologies associated with regions of decreased chromatin accessibility include the ‘*I*-*Smad (inhibition of Smad) binding* (*GO:0070411)’*. I-Smads (inhibitory-Smads) are one of three sub-types of Smads that inhibit intracellular signalling of TGF-β by various mechanisms including receptor-mediated inhibition [94]. This coincides with the appearance of *‘Type 1 transforming growth factor beta receptor binding (GO: 0034713)’.* In addition, four genes (*SMAD2*, *DDX5*, *SMURF1* and *SMAD6*) are associated with closed chromatin and *‘R*-*Smad binding (GO: 0005814)’*, which are part of the R-Smad sub-family that regulates TGF-β signalling directly [95, 96]. TGF-β induces I-Smad expression, and has been hypothesised to be a central component of dysregulated fibrotic processes in *Chlamydia*-infected cells, provoking runaway positive feedback loops that generate excessive ECM deposition and proteolysis, potentially leading to inflammation and scarring [16].

We also identify 11 genes localised within the cellular component *‘Microtubule organising centre (GO: 0044450)’*. Dynein-based motor proteins have been shown to move the chlamydial inclusion via the internal microtubule network to the MTOC (microtubule-organising centre); the close proximity to the MTOC is thought to facilitate the transfer of host vesicular cargo to the chlamydial inclusion [97].

Two similar ontologies *‘Ubiquitin*-*like protein ligase binding (GO: 0044389)’* and *‘Ubiquitin protein ligase binding (GO: 0031625)’* are involved in ubiquitination and protein quality control. The eukaryotic ubiquitination modification marks proteins for degradation and regulates cell signalling of a variety of cellular processes, including innate immunity and vesicle trafficking [98]. The deposition of ubiquitin onto intracellular pathogens is a conserved mechanism found in a diverse range of hosts [99]. In *Chlamydia*, host cell ubiquitin systems can mark chlamydial inclusions for subsequent destruction [100] and there is emerging evidence that various *Chlamydia* species, using secreted effectors and other proteins, are able to subvert or avoid these host ubiquitination marks for intracellular survival [100, 101]. Our observation of decreased chromatin accessibility of numerous ubiquitination genes, further highlighting the complex role of ubiquitination in chlamydial infection.

### Identification of transcription factor motifs

Putative TFs were identified from enriched motifs within all significant differential chromatin-accessible regions at each time post-infection (Additional file 6). Eleven of the most significant TF motifs are shown in Table 1 and span the chlamydial developmental cycle. *IRF3* (Interferon Regulatory Factor) motifs are enriched at 1 hpi; *IRF3* is a key transcriptional regulator of type I interferon (IFN)-dependent innate immune responses and is induced by chlamydial infection. The type I IFN response to chlamydial infection can induce cell death or enhance the susceptibility of cells to pro-death stimuli [102], but may also be actively dampened by *Chlamydia* [103, 104]. Specificity Protein 1 (*Sp1*) is a zinc-finger TF that binds to a wide range of promoters with GC-rich motifs. *Sp1* may activate or repress transcription in a variety of cellular processes that include responses to physiological and pathological stimuli, cell differentiation, growth, apoptosis, immune responses, response to DNA damage and chromatin remodelling [105, 106].

**Table 1:** Motifs and enriched transcription factors

Time	Motif	*p* value	Target sequences with Motif (%)	Background sequences with Motif (%)	Transcription factor
1		1e−13	10.53	3.84	IRF3*
24		1e−12	17.45	9.78	Homeobox*
48		1e−28	7.67	1.82	Sp1(Zf)
		1e−22	6.30	1.58	KLF9(Zf)
		1e−21	7.58	2.40	KLF3(Zf)
		1e−15	32.58	23.46	MEF2C*
		1e−13	9.81	4.90	KLF6(Zf)
		1e−10	6.30	2.87	KLF10(Zf)
		1e−7	11.06	7.18	KLF5(Zf)
		1e−7	10.45	6.71	NFYB
		1e−6	5.37	2.87	E2F3

Target sequences are significant differential peaks and background sequences are randomly selected throughout the genome to determine significance. A star (*) denotes a de novo motif where various sources were used to annotate the corresponding transcription factor

The majority of TF motifs enriched at 48 h correspond to Krüppel-like-factors (KLFs). KLFs are zinc-finger TFs in the same family as *Sp1*, which is also enriched at 48 h. The members of this large family orchestrate a range of paracrine and autocrine regulatory circuits and are ubiquitously expressed in reproductive tissues [107]. Dysregulation of KLFs and their dynamic transcriptional networks is associated with a variety of uterine pathologies [107]. We find motif enrichment for five distinct KLFs (*KLF3*, *KLF5*, *KLF6*, *KLF9* and *KLF10*) at 48 h, in addition to further KLFs at 12 (*KLF3*, *KLF4*, *KLF6*, *KLF9*), 24 h (*KLF 10*) and 48 h (*KLF 4*), when relaxing the initial filtering steps (Additional file 6). *KLF5* is a transcriptional activator found in various epithelial tissues and is linked to regulation of inflammatory signalling, cell proliferation, survival and differentiation [108]. *KLF6* is also a transcriptional activator ubiquitously expressed across a range of tissues and plays a crucial role in regulating genes involved with tissue development, differentiation, cell cycle control, and proliferation [109]. Target genes include collagen α1, keratin 4, TGFβ type I and II receptors, and others [110]. *KLF3* is primarily associated as a strong transcriptional repressor associated with adipogenesis and lipid metabolism [111], with expression rates varying across different tissues and cell types [109]. *KLF9* and 10 also act as transcriptional repressors, but are ubiquitously expressed across a wider range of tissues [112]. *KLF9* is a tumour suppressor [113] and regulates inflammation, while *KLF10* has a major role in TGF-β-linked inhibition of cell proliferation, inflammation and initiating apoptosis [114].

Histone deacetylases (HDACs) modify the core histones of the nucleosome, providing an important function in transcriptional regulation [115], and many bacterial pathogens subvert HDACs to suppress host defences [15]. *KLF9* and *KLF10* share the co-factor *Sin3A* (SIN3 Transcription Regulator Family Member A) [112], which is also a core component of the chromatin-modifying complex mediating transcriptional repression [116]. The Sin3a/HDAC complex is made up of two histone deacetylases *HDAC1* and *HDAC2*. *HDAC2* has increased chromatin accessibility at all four time points, and *HDAC9* has increased chromatin accessibility at 1, 24 and 48 h, further supporting the potential for histone modifications to be a component of the host cell response to chlamydial infection, or to be targets of chlamydial effectors [17].

All enriched TFs (Table 1) were compared against gene expression studies to ensure that each TF is expressed in HEp2 and HeLa cells from similar times (data not shown). To compare each TFs regulatory control on target genes, we examined the genes underlying significant regions associated with each motif, comparing fold-change differences across different infection-based environments (Additional file 7). There is no consensus of gene expression associated with any of the enriched TFs. For example, previous studies have indicated that *KLF3* is a strong transcriptional repressor; here, we see a range of fold-changes in the *KLF3* regulated genes, with two of the three datasets examined showing only a slight decrease in mean expression (Additional file 7). We attribute these differences to the variability inherent to distinct infection models. Nevertheless, these results further highlight the diverse and complex mechanisms associated with the epithelial cell response to chlamydial infection.

## Conclusions

We describe comprehensive changes to chromatin accessibility upon chlamydial infection in epithelial cells in vitro using FAIRE-Seq. We identify both conserved and time-specific infection-responsive changes to a variety of features and regulatory elements over the course of the chlamydial developmental cycle that may shape the host cell response to infection, including promotors, enhancers, and transcription factor motifs. Some of these changes are associated with genomic features and genes known to be relevant to chlamydial infection, including innate immunity and complement, acquisition of host cell lipids and nutrients, intracellular signalling, cell–cell adhesion, metabolism and apoptosis.

Host cell chromatin accessibility changes are evident over the entire chlamydial developmental cycle, with a large proportion of all chromatin accessibility changes at 48 h post-infection. This likely reflects the confluence of late stages of developmental cycle events, however significant changes to chromatin accessibility are readily apparent as early as 1 h post-infection. We find altered chromatin accessibility in several gene regions, ontologies and TF motifs associated with ECM moieties, particularly cadherins and their interconnected regulatory pathways, and Smad signalling. Disruption of the ECM is thought to be a central component of dysregulated fibrotic processes that may underpin the inflammatory scarring outcomes of chlamydial infection [16], and our data further highlights a central role of the ECM in epithelial cell responses to infection. We also identify factors that have not been previously described in the context of chlamydial infection, notably the enrichment of the KLF family of transcription factor motifs within differential chromatin-accessible regions in the latter stages of infection. Dysregulation of the biologically complex KLFs and their transcriptional networks is linked to several reproductive tract pathologies in both men and women [107], thus our discovery of enriched KLF-binding motifs in response to chlamydial infection is compelling, given the scale and burden of chlamydial reproductive tract disease globally [3].

In summary, this is the first genome-scale analysis of the impact of chlamydial infection on the human epithelial cell epigenome, encompassing the chlamydial developmental cycle at early, mid and late times. This has yielded a novel perspective of the complex host epithelial cell response to infection, and will inform further studies of transcriptional regulation and epigenomic regulatory elements in *Chlamydia*-infected human cells and tissues. Examination of the multifaceted human epigenome, and its potential subversion by *Chlamydia*, using in vivo mouse models of infection and ex vivo human reproductive tract tissues, will continue to shed light on how the host cell response contributes to infection outcomes.

## Supplementary information

**Additional file 1.** Summary of mapped reads by time and condition. Table summarising the number of mapped reads for each time and infection condition.

**Additional file 2.** Genome coverage plots. Significant peaks from each replicate as determined by MACS2. Screenshots are from IGV (Integrative Genomics Viewer) showing that all replicates contain significant peaks genome-wide (human genome) without any visual chromosomal bias.

**Additional file 3.** Annotation of all significant peaks. Annotation of all the significant peaks, with tabs separating genomic features and fold-change regulation.

**Additional file 4.** Conserved transcription factor expression. Motifs associated with each transcription factor (TF) as identified within the conserved regions. Genes associated with these regions were compared against relevant gene expression data to identify their level of regulation during infection. The TF *POU3F2* was not able to be compared as the motif was only identified within intergenic regions that could not be overlapped. **A)** *ETS1* TF. **B)** *TFAP4* TF. **C)** *PKNOX1* TF.

**Additional file 5.** Time specific regions. The list of time-specific differential chromatin-accessible regions. It should be noted that some genes in these lists are repeated at each time due to multiple peaks occurring at an annotated interval, that enhancers can affect more than one gene, and single genes can be affected by more than one enhancer.

**Additional file 6.** Complete list of motifs and transcription factors. The complete list of significant motifs and enriched transcription factors.

**Additional file 7.** Time-specific transcription factor expression. Motifs associated with each transcription factor (TF) (Table 1) were identified within significant differentially accessible regions. Genes associated with these regions were compared against relevant gene expression data to identify their level of regulation during infection. **A)** *IRF3* TF from 1 h. **B)** *Homeobox* TF from 24 h. **C-K)** Nine TFs identified at 48 h.
