# Supplementary figures and images for: Chromatin accessibility dynamics of Chlamydia-infected epithelial cells
Source: Epigenetics Chromatin. 2020 Oct 27;13:45. doi: 10.1186/s13072-020-00368-2 (PMC7590614; doi:10.1186/s13072-020-00368-2)

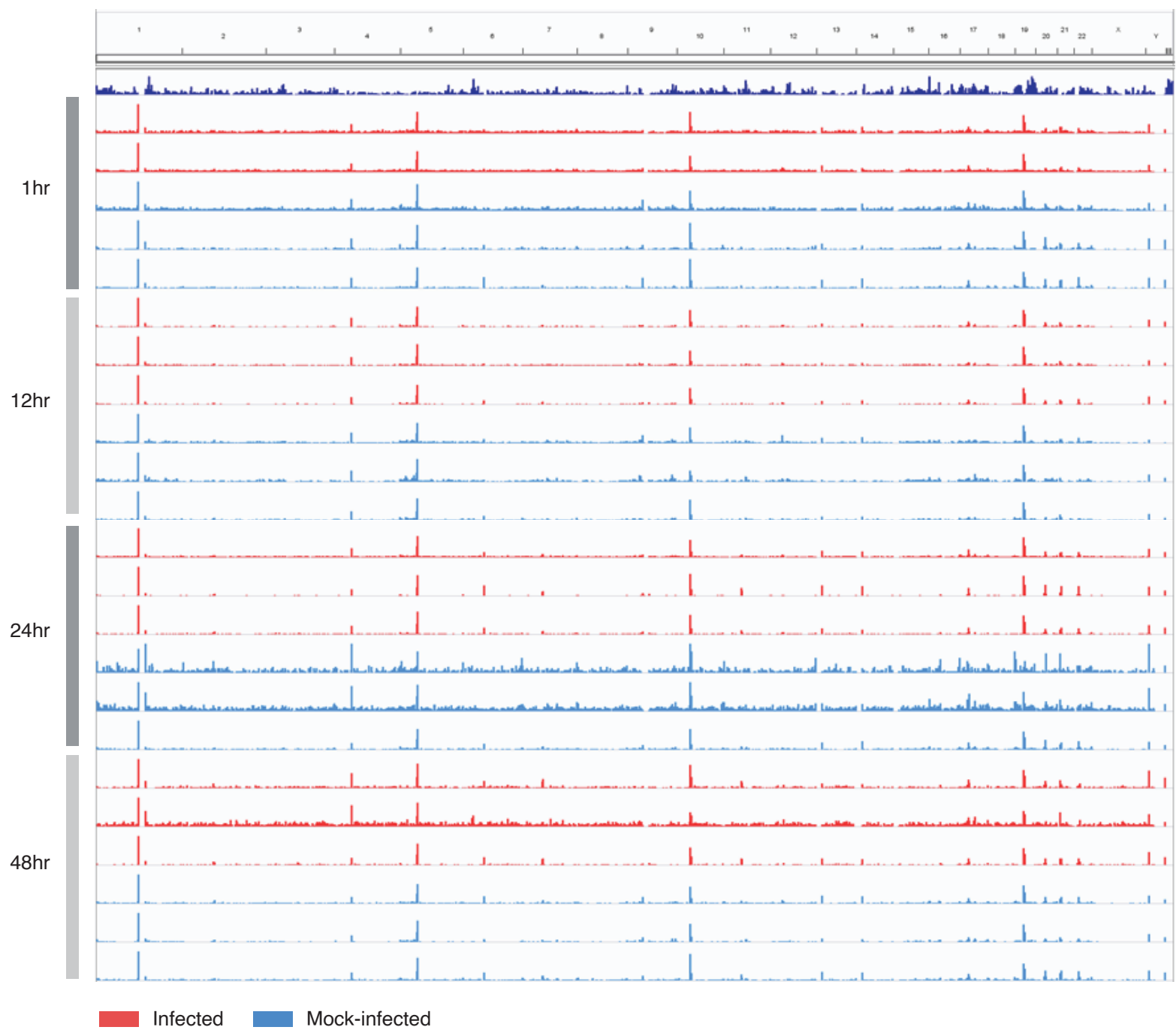

Supplement: Supplementary file 2 — Additional file 2. Genome coverage plots. Significant peaks from each replicate as determined by MACS2. Screenshots are from IGV (Integrative Genomics Viewer) showing that all replicates contain significant peaks genome-wide (human genome) without any visual chromosomal bias. [file 13072_2020_368_MOESM2_ESM.pdf]

**A)****EFT1**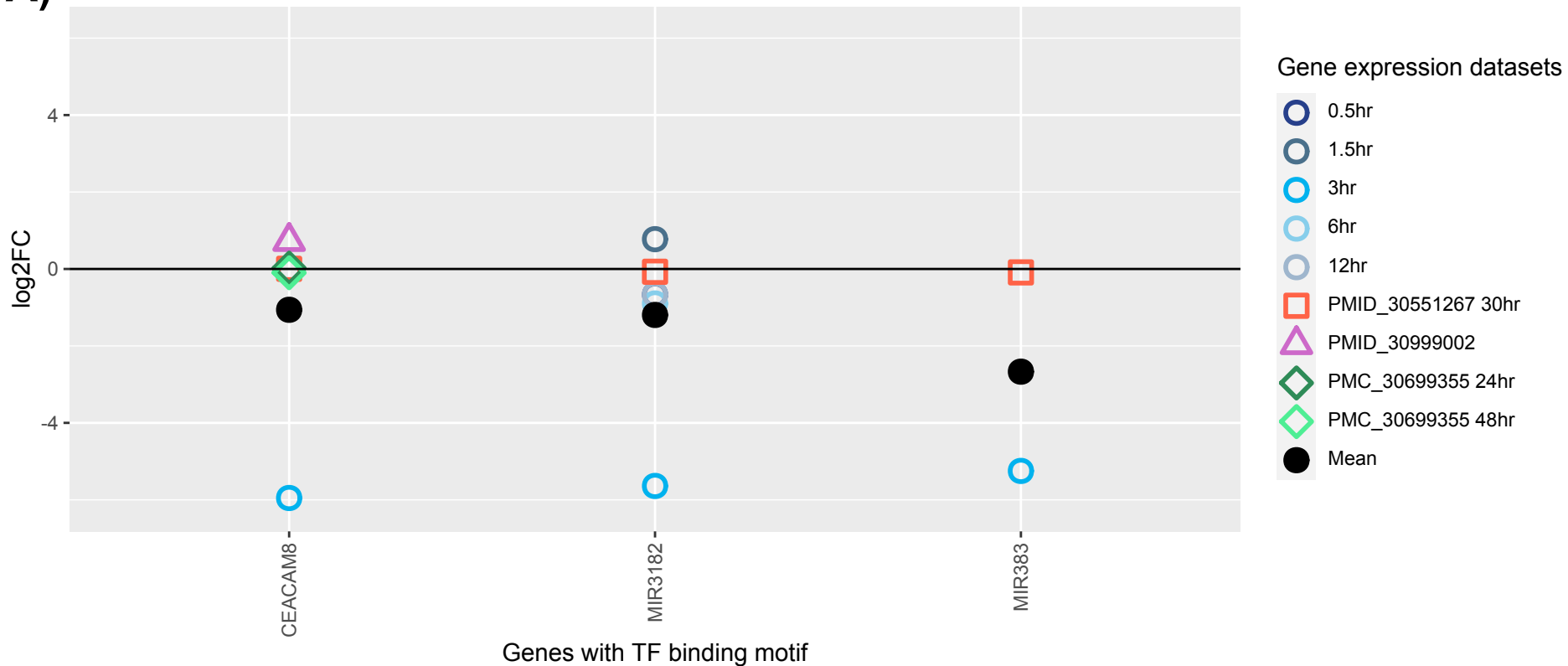

**B)****TFAP4**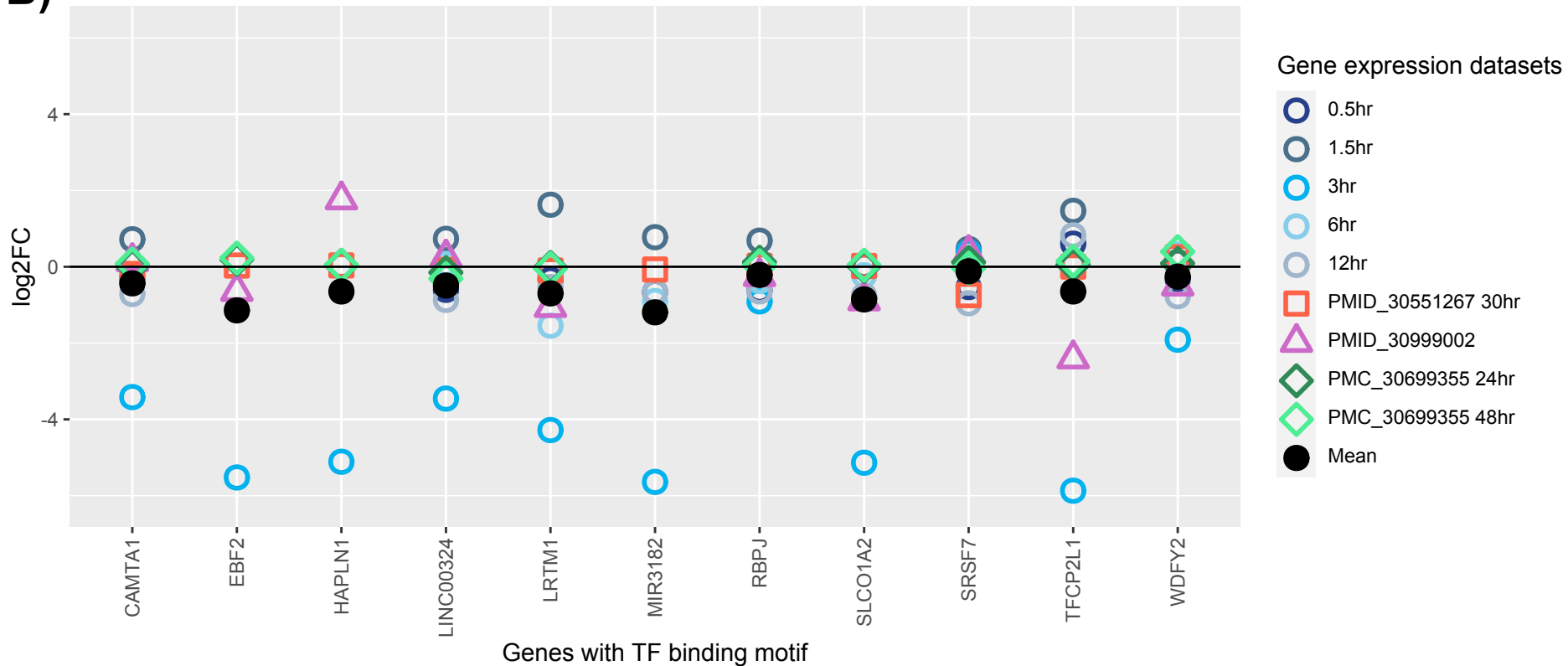

**c)**

PKNX1

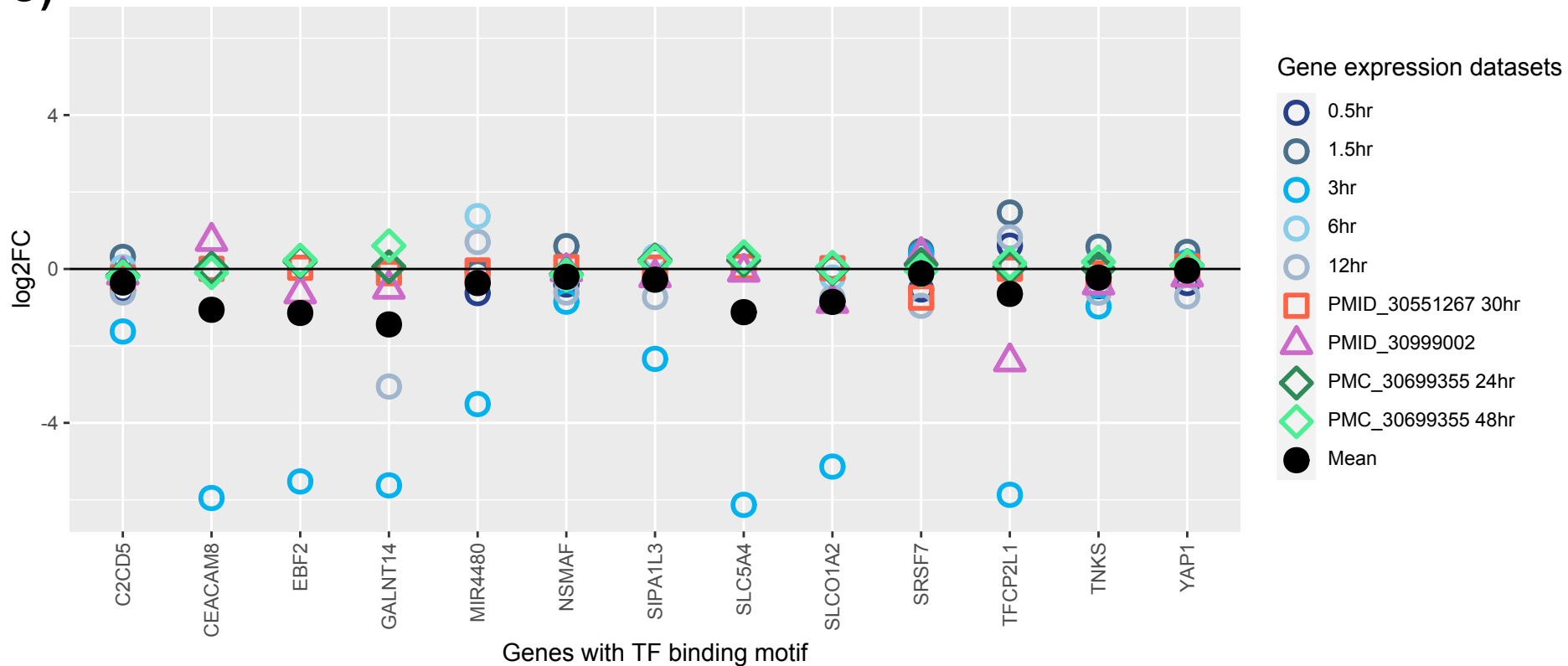

Supplement: Supplementary file 4 — Additional file 4. Conserved transcription factor expression. Motifs associated with each transcription factor (TF) as identified within the conserved regions. Genes associated with these regions were compared against relevant gene expression data to identify their level of regulation during infection. The TF POU3F2 was not able to be compared as the motif was only identified within intergenic regions that could not be overlapped. A) ETS1 TF. B) TFAP4 TF. C) PKNOX1 TF. [file 13072_2020_368_MOESM4_ESM.pdf]
