# Supplementary material for: Chromatin accessibility dynamics of Chlamydia-infected epithelial cells
Source: Epigenetics Chromatin. 2020 Oct 27;13:45. doi: 10.1186/s13072-020-00368-2 (PMC7590614; doi:10.1186/s13072-020-00368-2)
Supplement: Supplementary file 7 — Additional file 7. Time-specific transcription factor expression. Motifs associated with each transcription factor (TF) (Table 1) were identified within significant differentially accessible regions. Genes associated with these regions were compared against relevant gene expression data to identify their level of regulation during infection. A) IRF3 TF from 1 h. B) Homeobox TF from 24 h. C-K) Nine TFs identified at 48 h. [file 13072_2020_368_MOESM7_ESM.pdf]

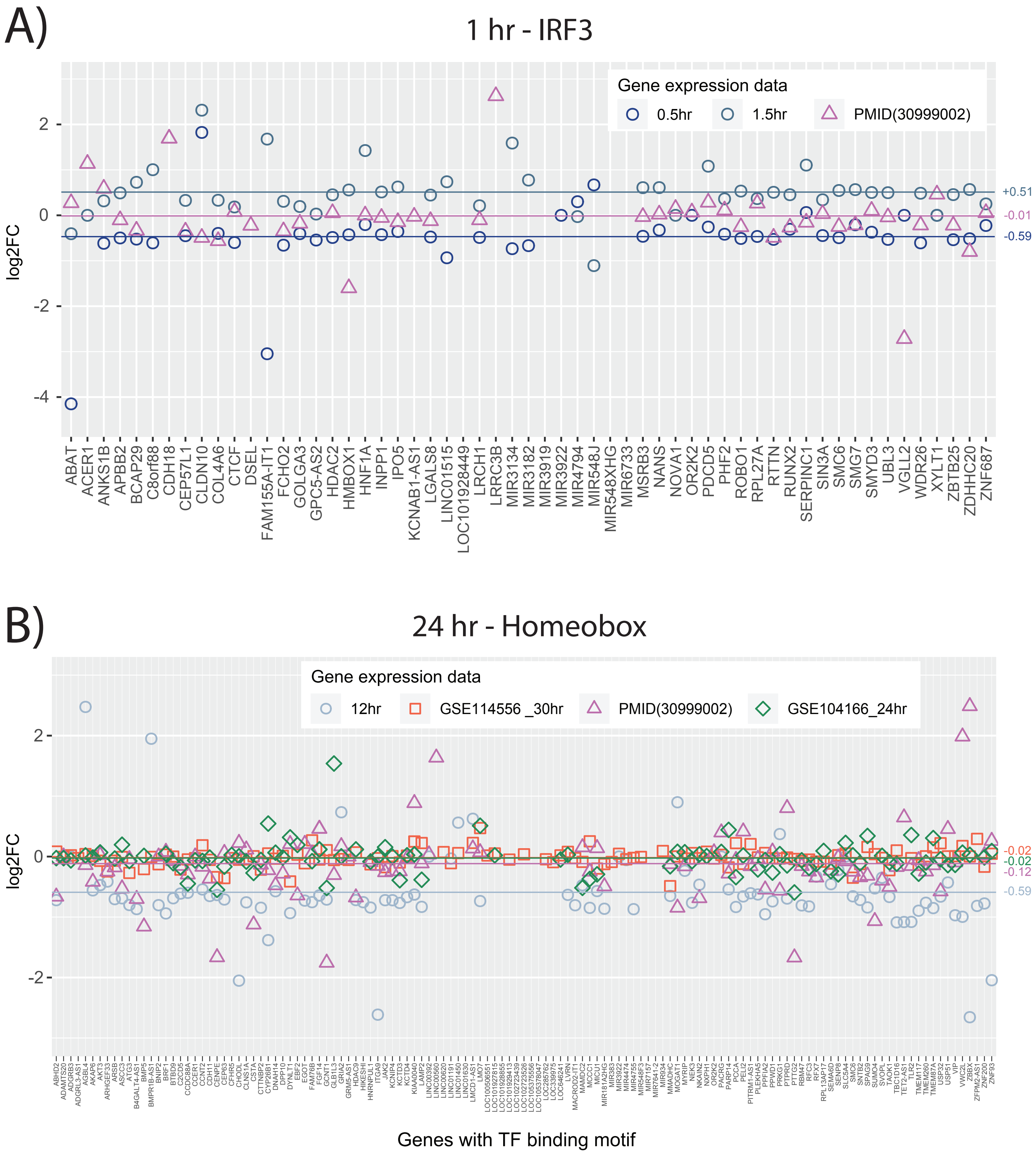
A)
1 hr - IRF3
Gene expression data
0.5hr
1.5hr
PMID(30999002)
log2FC
2
0
-2
-4
+0.51
-0.01
-0.59
ABAT
ACER1
ANKS1B
APBB2
BCAP29
C8orf88
CDH18
CEP57L1
CLDN10
COL4A6
CTCF
DSEL
FAM155A-IT1
FCHO2
GOLGA3
GPC5-AS2
HDAC2
HMBOX1
HNF1A
INPP1
IPO5
KCNAB1-AS1
LGALS8
LINC01515
LOC101928449
LRCH1
LRRC3B
MIR3134
MIR3182
MIR3919
MIR3922
MIR4794
MIR548J
MIR548XHG
MIR6733
MSRB3
NANS
NOVA1
OR2K2
PDCD5
PHF2
ROBO1
RPL27A
RTTN
RUNX2
SERPINC1
SIN3A
SMC6
SMG7
SMYD3
UBL3
VGLL2
WDR26
XYLT1
ZBTB25
ZDHHC20
ZNF687
B)
24 hr - Homeobox
Gene expression data
12hr
GSE114556 _30hr
PMID(30999002)
GSE104166_24hr
log2FC
2
0
-2
-0.02
-0.02
-0.12
-0.59
Genes with TF binding motif

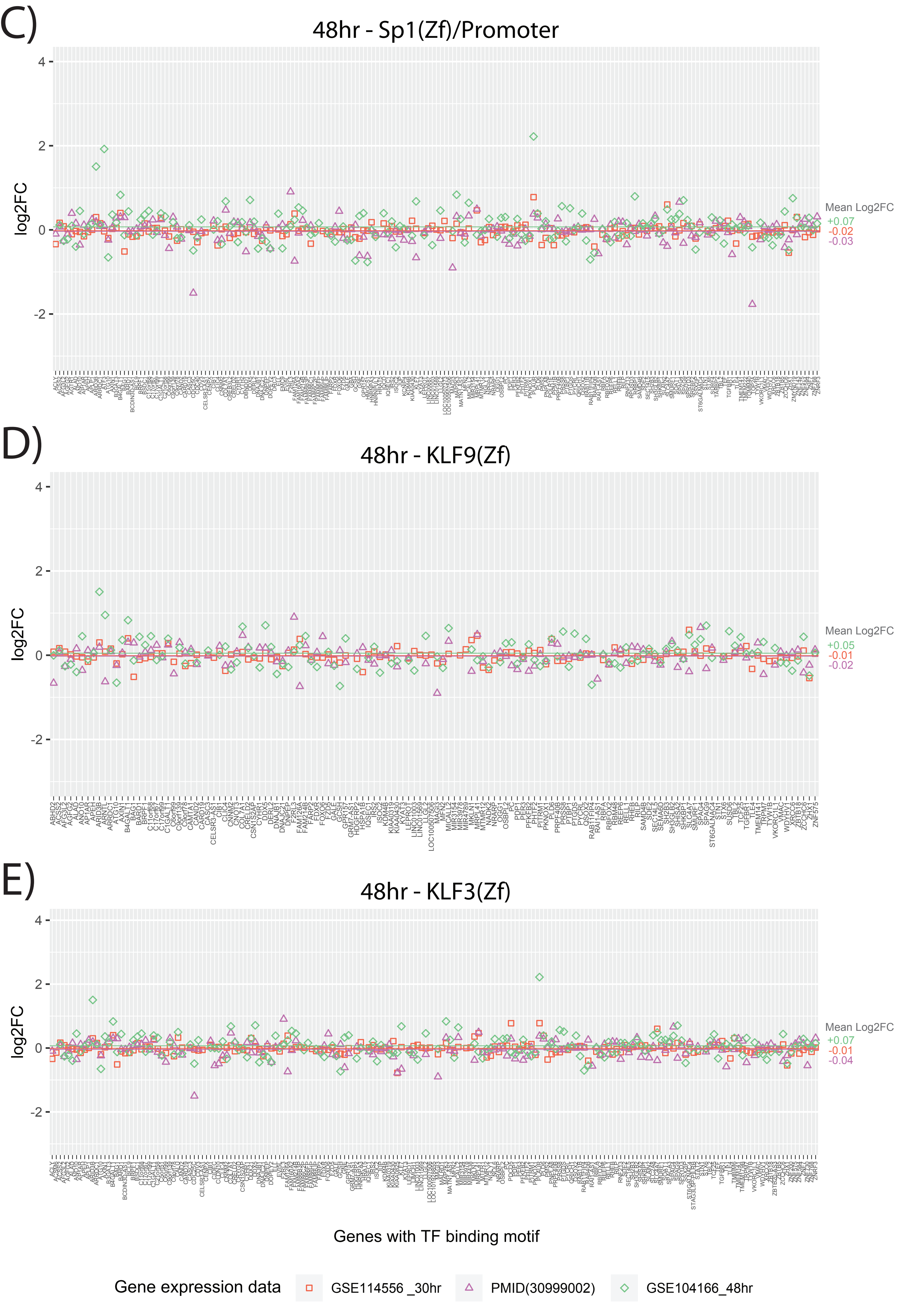
C)
48hr - Sp1(Zf)/Promoter
log2FC
Mean Log2FC
+0.07
-0.02
-0.03
D)
48hr - KLF9(Zf)
log2FC
Mean Log2FC
+0.05
-0.01
-0.02
E)
48hr - KLF3(Zf)
log2FC
Mean Log2FC
+0.07
-0.01
-0.04
Genes with TF binding motif
Gene expression data
GSE114556 _30hr
PMID(30999002)
GSE104166_48hr

F)

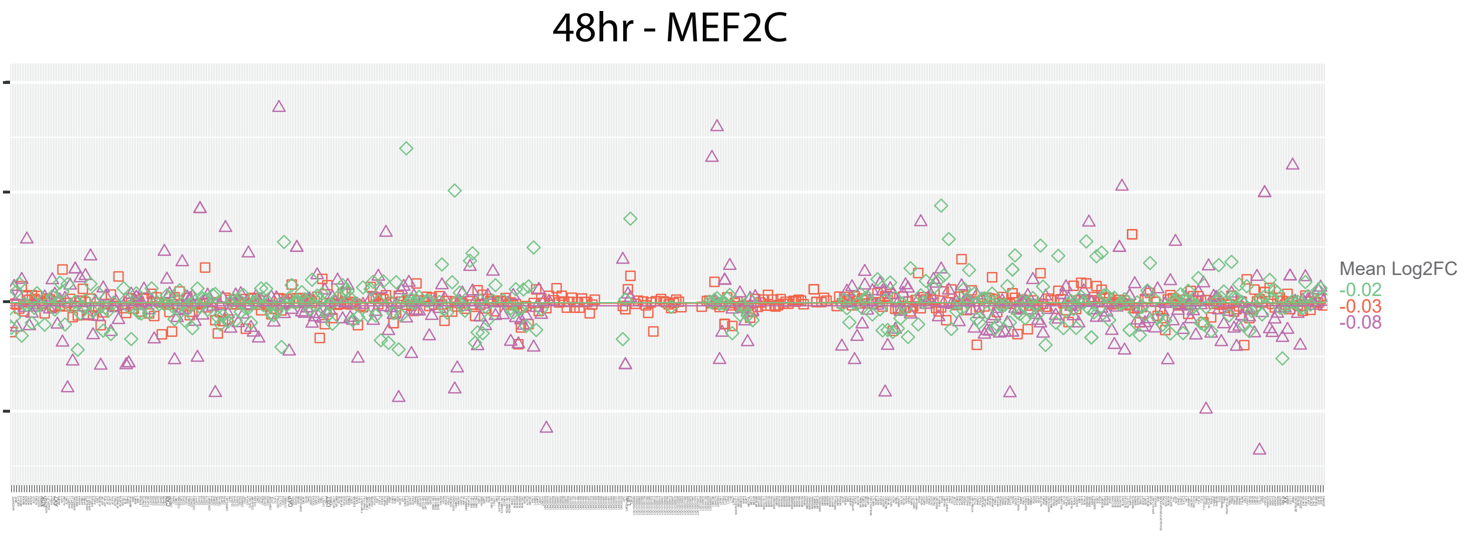
48hr - MEF2C
log2FC
4
2
0
-2
Mean Log2FC
-0.02
-0.03
-0.08

G)

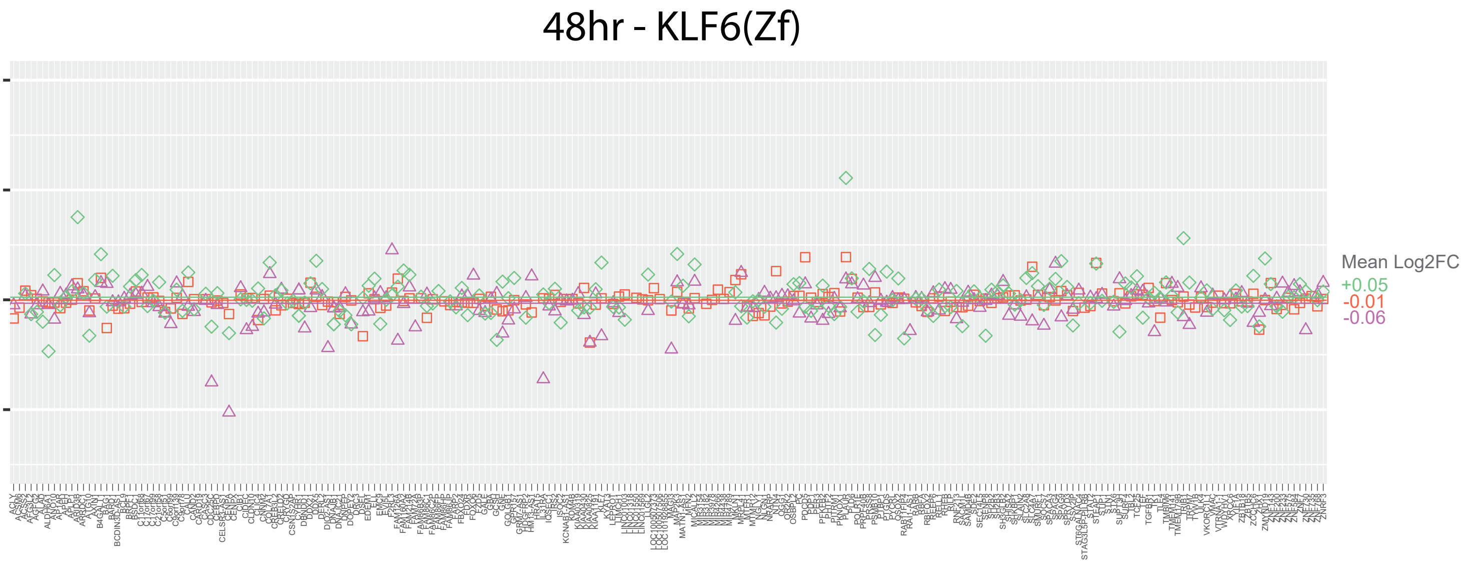
48hr - KLF6(Zf)
log2FC
4
2
0
-2
Mean Log2FC
+0.05
-0.01
-0.06

H)

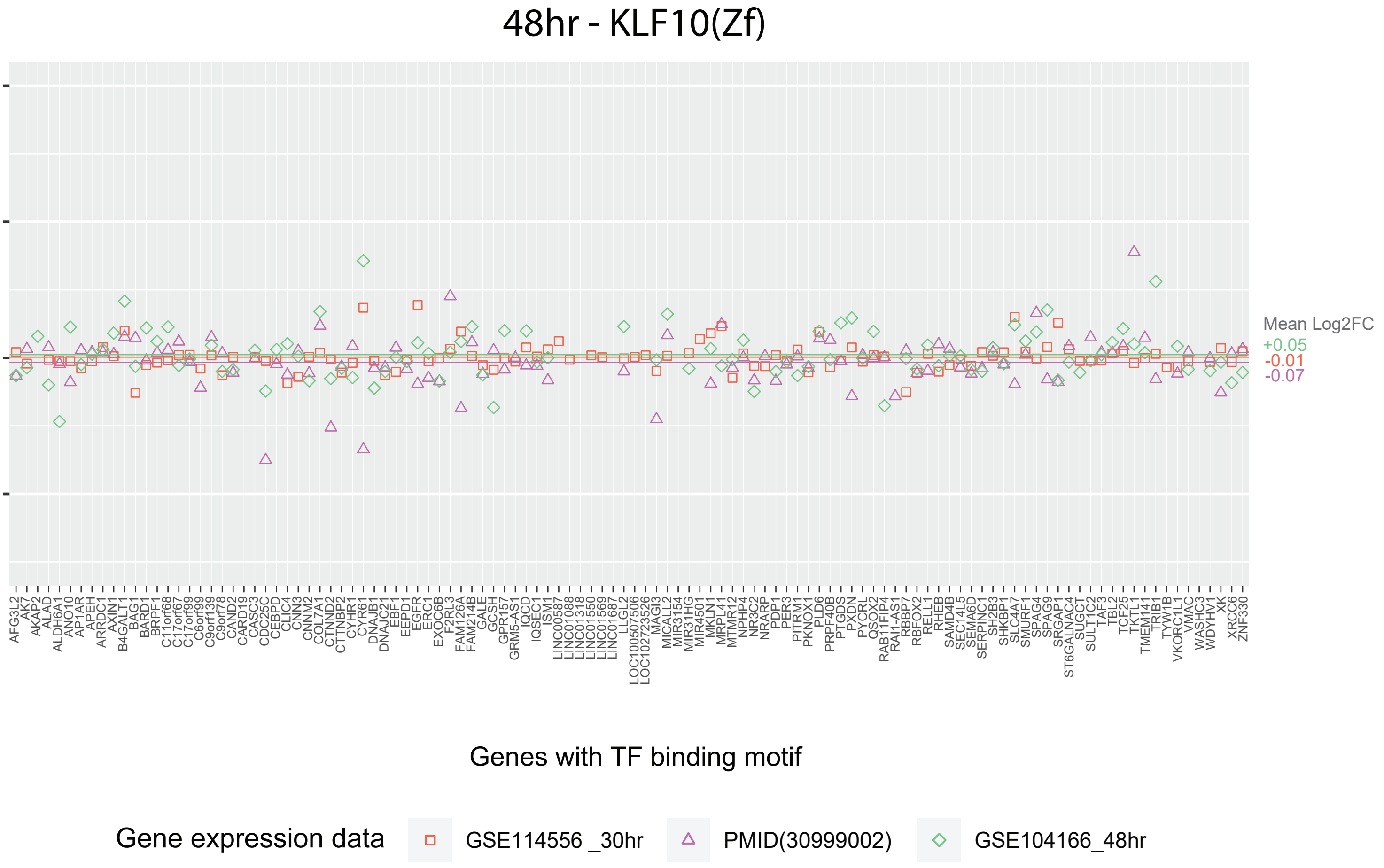
48hr - KLF10(Zf)
log2FC
4
2
0
-2
Mean Log2FC
+0.05
-0.01
-0.07
AFG3L2
AK7
AKAP2
ALAD
ALDH6A1
ANO10
AP1AR
APEH
ARRDC1
AXIN1
B4GALT1
BAG1
BARD1
BRPF1
C11orf68
C17orf67
C17orf99
C6orf99
C9orf139
C9orf78
CAND2
CARD19
CASC3
CDC25C
CEBPD
CLIC4
CNN3
CNNM2
COL7A1
CTNND2
CTTNBP2
CYHR1
CYR61
DNAJB1
DNAJC21
EBF1
EEPD1
EGFR
ERC1
EXOC6B
F2RL3
FAM126A
FAM214B
GALE
GCSH
GPR157
GRM5-AS1
IQCD
IQSEC1
ISM1
LINC00587
LINC01088
LINC01318
LINC01550
LINC01569
LINC01687
LLGL2
LOC100507506
LOC102723526
MAGI3
MICALL2
MIR3154
MIR31HG
MIR4501
MKLN1
MRPL41
MTMR12
NPHP4
NR3C2
NRARP
PDP1
PER3
PITRM1
PKNOX1
PLD6
PRPF40B
PTGDS
PXDN
PYCRL
QSOX2
RAB11FIP4
RAI1-AS1
RBBP7
RBFOX2
RELL1
RHEB
SAMD4B
SEC14L5
SEMA6D
SERPINC1
SH2B3
SHKBP1
SLC4A7
SMURF1
SPAG4
SPAG9
SRGAP1
ST6GALNAC4
SUGCT
SULT1C2
TAF3
TBL2
TCF25
TKTL1
TMEM141
TRIB1
TYW1B
VKORC1L1
VMAC
WASHC3
WDYHV1
XK
XRCC6
ZNF330
Genes with TF binding motif
Gene expression data
GSE114556 _30hr
PMID(30999002)
GSE104166_48hr

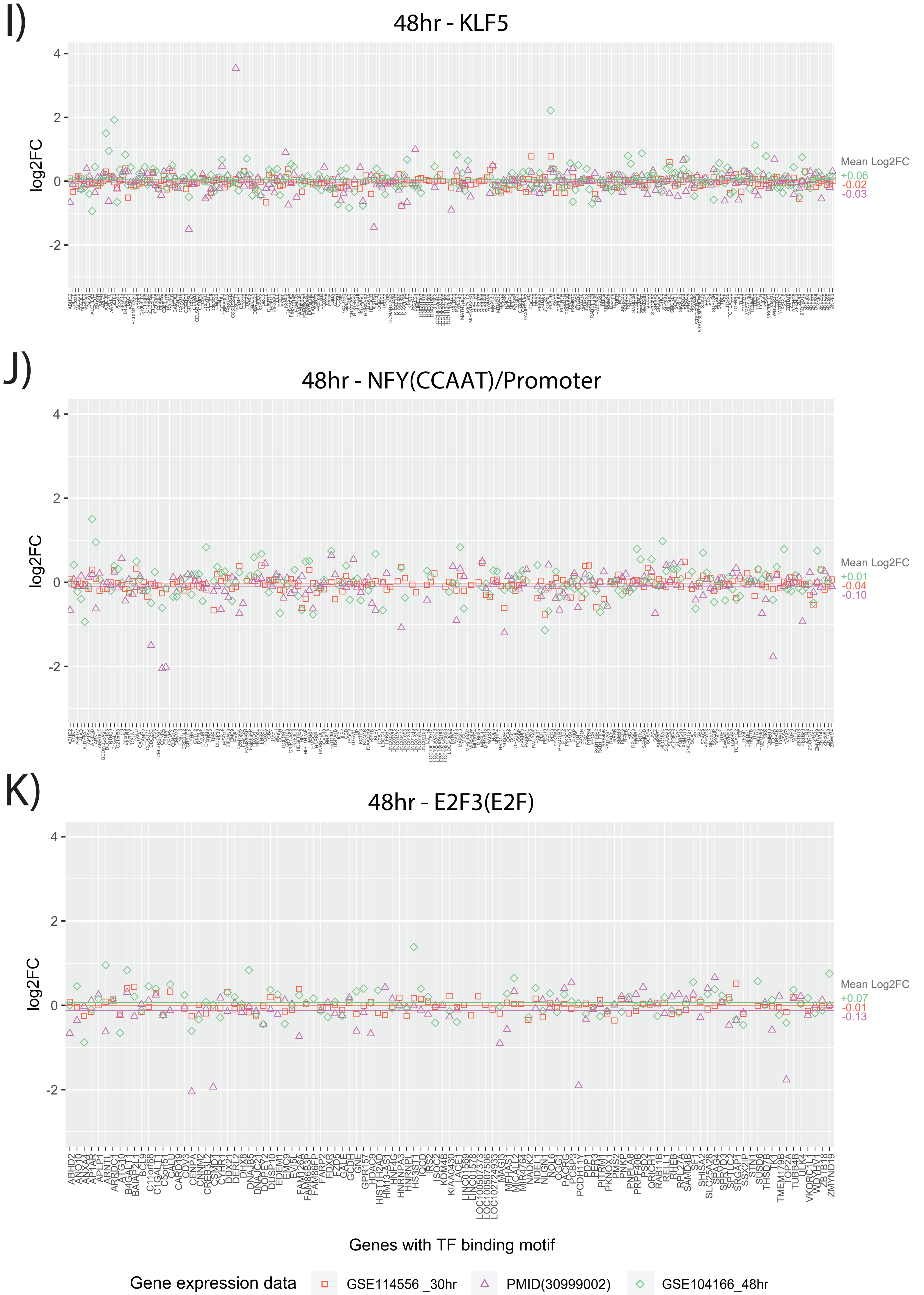
I)
48hr - KLF5
log2FC
4
2
0
-2
Mean Log2FC
+0.06
-0.02
-0.03
J)
48hr - NFY(CCAAT)/Promoter
log2FC
4
2
0
-2
Mean Log2FC
+0.01
-0.04
-0.10
K)
48hr - E2F3(E2F)
log2FC
4
2
0
-2
Mean Log2FC
+0.07
-0.01
-0.13
Genes with TF binding motif
Gene expression data
GSE114556 _30hr
PMID(30999002)
GSE104166_48hr
